# Calreticulin regulates a switch between osteoblast and chondrocyte lineages derived from murine embryonic stem cells

**DOI:** 10.1074/jbc.RA119.011029

**Published:** 2020-03-27

**Authors:** Carlos Pilquil, Zahra Alvandi, Michal Opas

**Affiliations:** Department of Laboratory Medicine and Pathobiology, University of Toronto, Toronto, Ontario M5S 1A8, Canada

**Keywords:** embryonic stem cell (ESC), glycogen synthase kinase 3 (GSK-3), Wnt signaling, chondrogenesis, NFAT transcription factor, calreticulin, osteogenesis, nuclear factor of activated T cells 1 (NFATC1), gene regulation, cell fate decision, development

## Abstract

Calreticulin is a highly conserved, ubiquitous Ca^2+^-buffering protein in the endoplasmic reticulum that controls transcriptional activity of various developmental programs and also of embryonic stem cell (ESC) differentiation. Calreticulin activates calcineurin, which dephosphorylates and induces the nuclear import of the osteogenic transcription regulator nuclear factor of activated T cells 1 (NFATC1). We investigated whether calreticulin controls a switch between osteogenesis and chondrogenesis in mouse ESCs through NFATC1. We found that in the absence of calreticulin, intranuclear transport of NFATC1 is blocked and that differentiation switches from osteogenic to chondrogenic, a process that could be mimicked by chemical inhibition of NFAT translocation. Glycogen synthase kinase 3β (GSK3β) deactivation and nuclear localization of β-catenin critical to osteogenesis were abrogated by calreticulin deficiency or NFAT blockade. Chemically induced GSK3β inhibition bypassed the calreticulin/calcineurin axis and increased osteoblast output from both control and calreticulin-deficient ESCs, while suppressing chondrogenesis. Calreticulin-deficient ESCs or cells treated with an NFAT blocker had enhanced expression of dickkopf WNT-signaling pathway inhibitor 1 (*Dkk1*), a canonical Wnt pathway antagonist that blocks GSK3β deactivation. The addition of recombinant mDKK1 switched osteogenic ESC differentiation toward chondrogenic differentiation. The results of our study indicate a role for endoplasmic reticulum calcium signaling via calreticulin in the differentiation of ESCs to closely associated osteoblast or chondrocyte lineages.

## Introduction

Calreticulin is a highly conserved, ubiquitous high-capacity Ca^2+^-buffering protein of the endoplasmic reticulum (ER)^3^ that is centrally located in a signaling network within the ER lumen (1). Calreticulin is uniquely endowed for this role as it controls ER [Ca^2+^] and the amount of Ca^2+^ releasable from the ER (2). Calreticulin-mediated ER signaling is poised to regulate transcriptional activity of developmental programs in general, and ESC differentiation in particular (3, 4). An important part of the transcriptional circuitry is the Ca^2+^/calmodulin-dependent phosphatase, calcineurin (5). Calreticulin regulates the release of Ca^2+^ from the ER required for activation of calcineurin (6), hence modulating the activity of several downstream genes that affect developmental processes (5). For example, ablation of calreticulin in mice is embryonic lethal due to faulty cardiomyogenesis (7), but it can be rescued in calreticulin-deficient (*crt*^−/−^) mice by specific heart-targeted overexpression of constitutively activated calcineurin (8). Overexpression of calcineurin in mice enhances osteoblast differentiation and bone formation (9), whereas calcineurin-null mice have diminished bone formation and severe osteoporosis, due to reduced osteoblast differentiation (9). Treatment of adult mice with the calcineurin inhibitor, FK506, reduces bone mass by primarily blocking osteoblastic bone formation (10). Calcineurin affects osteoblastogenesis by dephosphorylating transcription factors called the cytoplasmic nuclear factors of activated T cells (NFATC), thereby allowing their entry into the nucleus for gene regulation (5). NFATC1-deficient mice have defective bone formation, whereas mice with constitutively active nuclear NFATC1 in osteoblasts have increased osteoblast proliferation, high bone mass, and up-regulation of genes that promote osteogenic Wnt pathways (11).

Presently, there is substantial evidence for the role of calcineurin/NFAT in bone formation, but little for chondrocyte determination. Osteoblast and chondrocyte cell lineages are coupled during the nascent skeletal formation process known as endochondral ossification (12) and associated spatially in bone and cartilage of the mature skeleton, which requires a coordinated mechanism to specify the distinct lineages during development. ESCs can be differentiated to produce bone and chondrocyte cell lineages through the combination of defined media and the addition of bioactive signaling proteins and molecules (13). The present study focuses on the ER Ca^2+^-signaling role of calreticulin via the calcineurin/NFATC1 axis and GSK3β-deactivation in the differentiation to either osteoblasts or chondrocytes from murine ESCs. We found that calreticulin affects the fate in differentiating ESCs by determining NFATC1 loci of residence. Calreticulin suppresses the expression of the chondrogenic master transcription factor, *Sox9*, thereby suppressing the specification of chondrocytes, while simultaneously permitting osteoblast specification. Lineage determination is also accomplished by deactivation of GSK3β, which we show to be regulated by calreticulin and its downstream effector, NFAT. The absence of calreticulin and intranuclear NFAT favors chondrocyte specification despite the cells being cultured under an osteogenic differentiation protocol. Collectively, we describe a novel mechanism by which ER-Ca^2+^ signaling determines lineage specification to either an osteoblast or chondrocyte lineage in a mutually exclusive manner.

## Results

### Calreticulin regulates osteoblasts versus chondrocyte lineage specification

**Figure 1.**
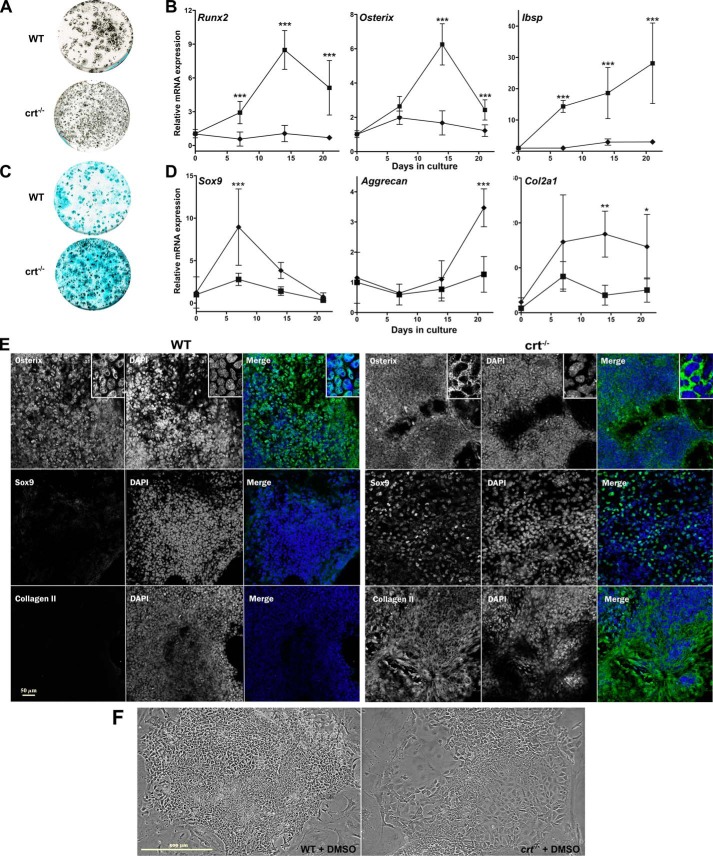
**Calreticulin affects specification of osteoblasts and chondrocytes from ESCs.** WT control ESCs and *crt*^−/−^ ESCs were differentiated using an osteogenic protocol and evaluated for Ca^2+^-based mineralization with von Kossa stain at day 26 (*A*) or for acidic proteoglycans secretion using Alcian blue stain on day 21 (*C*). *B* and *D*, real-time quantitative RT-PCR (RT-qPCR) analysis of osteoblast marker genes (*B*) or chondrocyte markers (*D*) of WT (■) and *crt*^−/−^ (♦) ESCs through time of osteoblast differentiation. *E*, confocal images of immunofluorescence localization analysis using osterix, SOX9, or type II collagen antibody at day 19 of the osteogenic differentiation protocol of WT control ESCs or *crt*^−/−^ ESCs. Dual-channel images of a single field are displayed with protein of interest *labeled* in the *left panel*, DAPI-stained nuclei in the *right panel*, and the *RGB panel* as a *merged image* of *green* (protein of interest) and *blue* (DAPI). *Scale bar*, 50 μm. *F*, phase-contrast images of day 10 differentiated WT or *crt*^−/−^ cells. *Scale bar*, 500 μm. Data are expressed as means ± S.D. (*error bars*), *n* ≥ 3, and two-way ANOVA between WT and *crt*^−/−^ ESCs results (*B* and *D*): *Runx2* (*p* < 0.0001, *F* = 158.7); osterix (*p* < 0.0001, *F* = 111.7); *Ibsp* (*p* < 0.000.1, *F* = 108.9); *Sox9* (*p* < 0.0001, *F* = 20.84); aggrecan (*p* < 0.0002, *F* = 19.51), *Col2a1* (*p* = 0.0001, *F* = 20.63). Bonferroni post hoc analysis was as indicated: *, *p* < 0.05; **, *p* < 0.01; ***, *p* < 0.001.

**Figure 2.**
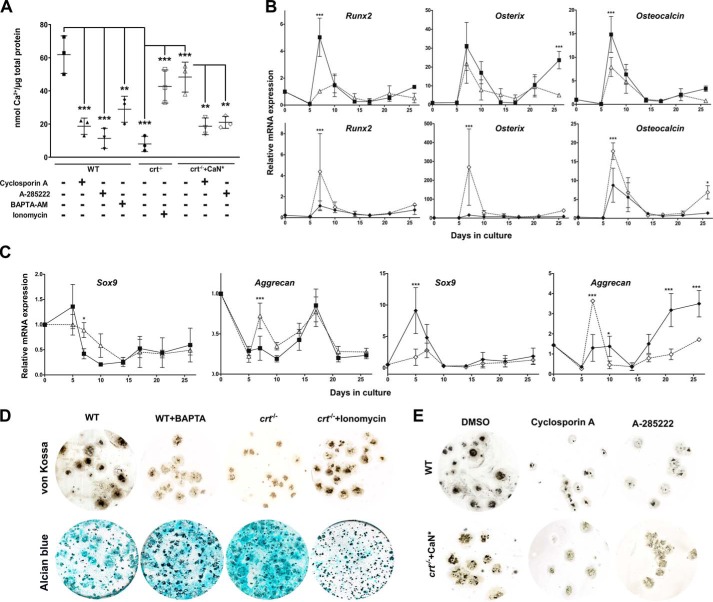
**Calreticulin/Ca^2+^, calcineurin, and NFAT regulates osteoblast and chondrocyte specification.** Differentiating WT control, *crt*^−/−^, and *crt*^−/−^+CaN* ESC cultures were treated on days 3–5 with cyclosporin A, BAPTA-AM, and ionomycin or on days 6–17 with A-285222, and then Ca^2+^-based mineralization was measured on day 21 by an Arsenazo III assay (*A*) or von Kossa (*D* and *E*). Acidic proteoglycans were stained with Alcian blue where indicated (*D*). *B* and *C*, RT-qPCR analysis of osteoblast (*B*) and chondrocyte (*C*) markers using RNA extracted at the indicated time points during the osteogenic differentiation protocol. WT (■) ESCs were treated with BAPTA/AM (▵), and *crt*^−/−^ (♦) ESCs were treated with ionomycin (♢) between days 3 and 5. Data are expressed as means ± S.D. (*error bars*), and *n* ≥ 3. *A*, one-way ANOVA result: *p* < 0.0001, *F* = 18.29; *B* and *C*, two-way ANOVA: transcript levels in WT control ESCs treated or not with BAPTA-AM (*Runx2, p* = 0.0002, *F* = 17.85; osterix, *p* = 0.0054, *F* = 8.815; osteocalcin, *p* = 0.0003, *F* = 16.21; *Sox9*, *p* = 0.5169, *F* = 0.4296; aggrecan, *p* = 0.0173, *F* = 6.261); transcript levels in *crt*^−/−^ ESCs treated or not with ionomycin (*Runx2*, *p* = 0.0462, *F* = 4.257; osterix, *p* = 0.0079, *F* = 7.931; osteocalcin, *p* = 0.0004, *F* = 15.07; *Sox9*, *p* = 0.0001, *F* = 18.65; aggrecan, *p* = 0.0003, *F* = 16). Bonferroni post hoc analysis was as indicated (*A–C*): *, *p* < 0.05; **, *p* < 0.01; ***, *p* < 0.001.

To characterize a role for calreticulin in osteoblastogenesis, we compared differentiation of calreticulin-deficient (*crt*^−/−^) ESCs to WT control ESCs. Mineralization was substantially reduced in *crt*^−/−^ cultures compared with the WT cells (Figs. 1*A* and 2 (*A* and *D*)). The increase in osteoblast activity of *crt*^+/+^ WT cells during differentiation was reflected in significantly higher transcript levels of osteoblast-specific genes *Runx2*, *Sp7* (osterix), and *Ibsp* in WT cells *versus crt*^−/−^ ESCs (Fig. 1*B*). The osteogenic transcription factor, osterix, is overwhelmingly located in the nuclei of the WT cultures at day 19 (Fig. 1*E*). Transcripts for Wnt signaling genes important for osteogenesis, including *Wnt10b* and *Wnt11* (14), were also elevated by the first week, but not in calreticulin-deficient ESCs (Fig. S1). Conversely, the anti-osteogenic canonical Wnt signaling antagonist, *Dkk1* (15), is enhanced in differentiating *crt*^−/−^ cultures (Fig. S1) and indicates a divergence from osteoblast differentiation. Overall, the data confirm a favored osteoblast specification from WT ESCs *versus* those from *crt*-null ESCs in both signaling environment and function.

Chondrocyte generation is strongly coupled to osteoblasts via their common cell lineage ancestors (16); therefore, we examined the cell fate of differentiated *crt*^−/−^ ESCs cultured in conditions favoring osteoblasts. The absence of calreticulin permitted a switch of the ESC fate from osteogenic toward a chondrogenic fate, as demonstrated by enhanced Alcian blue staining of glycosaminoglycans secreted by chondrocytes (Fig. 1*C*). Increased expression of chondrocytic genes, such as type II collagen α1 and aggrecan, and, importantly, increased transcript and protein levels of the chondrocyte master transcription factor *Sox9* (Fig. 1*D* and Fig. S2 (*A* and *B*)) in *crt*^−/−^ cells *versus* WT cells confirmed this switch in fate. The permissive effect of calreticulin absence on the cell fate switch from osteoblast to chondrocyte is apparent in the disparate manners of cell packing: WT culture cell colonies are tightly packed, whereas in *crt*^−/−^ colonies, the cells are more spread with less defined colony edges, and colonies are much less cell-dense (Fig. 1*F*). There was also a shift in the abundance of nuclear SOX9 over nuclear osterix between WT and *crt*^−/−^ cells. Immunofluorescence images show that at day 19, *crt*^−/−^ cells express significantly lower nuclear osterix levels and higher levels of the chondrogenic transcription factor SOX9 in the nucleus. There is also a higher level of staining of the cartilage marker type II collagen compared with WT cells (Fig. 1*E*), demonstrating a shift to chondrocyte specification in the absence of calreticulin.

### A signaling axis comprising calreticulin/Ca^2+^/calcineurin promotes osteoblast specification over chondrocyte specification

Calreticulin, by being the most robust ER Ca^2+^ buffer, is critical for Ca^2+^ homeostasis; the absence of calreticulin reduces both ER-releasable and total [Ca^2+^], and the converse is true for calreticulin overexpression (17). Ca^2+^ content of mineralized deposits in WT cultures was ∼7-fold higher than that in the *crt*^−/−^ cultures (Fig. 2*A*), and chelation of intracellular [Ca^2+^] with BAPTA/AM caused a substantial decrease in WT cell mineralization (Fig. 2 (*A* and *D*), von Kossa plates). Decreased osteoblast activity in the presence of BAPTA/AM was confirmed with a decrease of mRNA abundance of the osteoblast markers *Runx2*, osterix, and osteocalcin (Fig. 2*B*). In contrast, increasing intracellular [Ca^2+^] with ionomycin restored mineralization of the nonosteogenic *crt*^−/−^ cell cultures (Fig. 2 (*A* and *D*), von Kossa plates). Again, mRNA levels of osteoblast markers in *crt*^−/−^ cell cultures treated with ionomycin are increased compared with vehicle-treated cells (Fig. 2*B*). These results strongly indicate that the role of calreticulin in osteoblastogenesis is through its Ca^2+^ homeostasis function. Concurrently, as BAPTA/AM decreased osteoblast activity of WT cell cultures, there was a modest increase in acidic proteoglycan staining with Alcian blue (Fig. 2*D*) along with a modestly enhanced expression of Sox9 and aggrecan mRNA (Fig. 2*C*) and SOX9 protein (Fig. S2, *C* and *D*) promptly detected after the time frame of treatment with the intracellular Ca^2+^ chelator (days 3–5). The inverse is more notable when *crt*^−/−^ cell cultures were treated with ionomycin and the early *Sox9* expression decreased (Fig. 2*C* and Fig. S2 (*C* and *D*)). Aggrecan mRNA levels decreased at later time points (Fig. 2*C*), along with decreased Alcian blue staining (Fig. 2*D*).

Ca^2+^ released by calreticulin activates calcineurin (18, 19); hence, we examined the involvement of the calreticulin/Ca^2+^/calcineurin axis in osteoblastogenesis. Calcineurin activity was low in *crt*^−/−^ cells when compared with WT ESCs (Fig. S3*A*). A stable transfection of nonosteogenic *crt*^−/−^ cells with constitutively active calcineurin (cell line denoted *crt*^−/−^+CaN*) restored calcineurin activity (Fig. S3*A*) and induced dramatically high mRNA levels of osteoblast markers during the early period of differentiation (day 7; Fig. S3*B*) that translated to a restoration of osteogenic potential similar to that of *crt*^+/+^ WT cells (Fig. 2, *A* and *E*). Predictably, cyclosporin A inhibited calcineurin activity (Fig. S3*A*), which also suppressed mineralization in both differentiating control and *crt*^−/−^+CaN* ESCs (Fig. 2, *A* and *E*).

### The calreticulin/Ca^2+^/calcineurin axis controls NFATC1 nucleocytoplasmic trafficking

**Figure 3.**
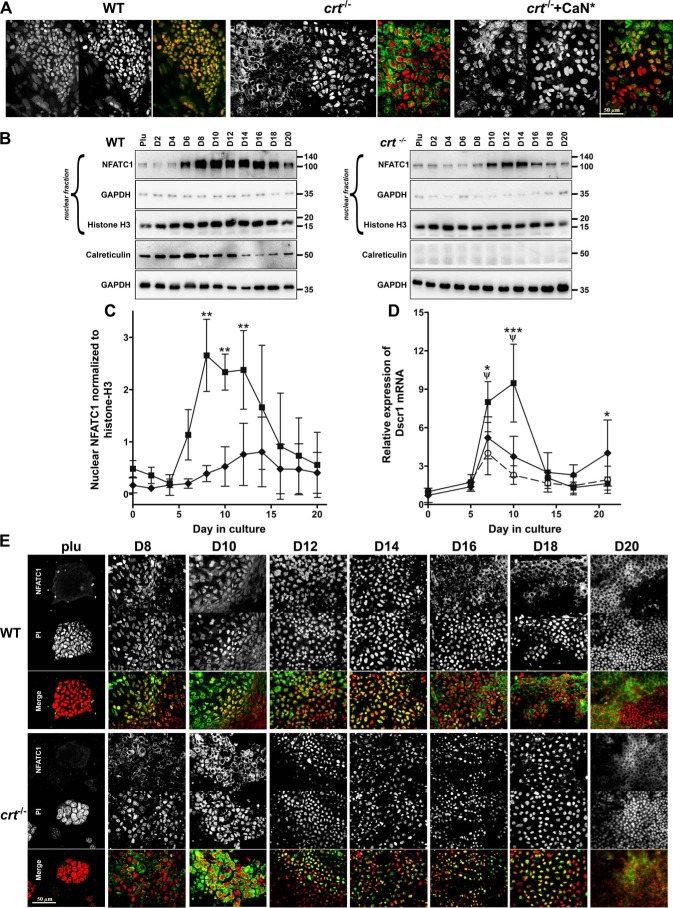
**Calreticulin regulates NFATC1 nucleocytoplasmic trafficking.** *A*, confocal immunofluorescence localization using NFATC1 antibody on WT control, *crt*^−/−^, and *crt*^−/−^+CaN* ESCs on day 10 of the osteoblast differentiation protocol. Dual-channel *grayscale images* of a single field are displayed with NFATC1-labeled cells in the *left panel*, PI-labeled nuclei in the *right panel*, and the *RGB panel* of a *merged image* of *green* (NFATC1) and *red* (PI) where *yellow* indicates overlap. *Scale bar*, 50 μm. *B*, Western blot analysis of nuclear fractions of WT control and *crt*^−/−^ ESCs undergoing osteoblast differentiation through time using NFATC1 antibodies and antibodies for GAPDH and histone H3 as cytosolic and nuclear markers, respectively. Shown are expression of calreticulin and (*below*) GAPDH from whole-cell lysates of WT and *crt*^−/−^ cells undergoing osteoblast differentiation. *C*, the graph is a quantitative representation of the density of the Western blotting bands of nuclear NFATC1 normalized to nuclear histone H3 of three experiments. Shown are WT (■) and *crt*^−/−^ (♦) ESCs. Shown is RT-qPCR analysis of the NFATC1 target gene *Dscr1* (*D*) through time of differentiation in DMSO-treated WT (■) or *crt*^−/−^ (♦) ESCs or WT ESCs treated with A-285222 (○) for days 6–17. Data are expressed as means ± S.D. (*error bars*), *n* ≥ 3; two-way ANOVA. *C*, *p* < 0.0001, *F* = 65.09. *D*, transcript levels of *Dscr1* between WT control and *crt*^−/−^ ESCs, *p* = 0.0304, *F* = 4.929; WT control ESCs treated or not with A285222, *p* < 0.0001, *F* = 47.5. Bonferroni post hoc analysis was as indicated (*C* and *D*): ***, *p* < 0.001; **, *p* < 0.01; *, *p* < 0.05; ψ, *p* < 0.001 for DMSO-treated WT *versus* A-285222–treated WT ESCs (*D*). *E*, confocal immunofluorescence localization analysis of NFATC1 trafficking in WT control and *crt*^−/−^ ESCs throughout the differentiation timeline. Dual-channel *grayscale images* of a single field are displayed with the *top rows* showing NFATC1 immunolocalization, PI-labeled nuclei in the *middle row*, and the *merged RGB images* in the *bottom rows. Scale bar*, 50 μm.

NFATC1 has been shown to be an effector for calreticulin and calcineurin to induce osteoblast specification (11, 20). Importantly, intranuclear localization of NFATC1 is significantly (>80% of cells) restored upon expression of constitutively active calcineurin in *crt*^−/−^+CaN* cells (Fig. 3*A*); therefore, akin to other systems, calcineurin dephosphorylates NFATC1 to promote its nuclear translocation. Immunofluorescence observations have been complemented by manual counts of NFATC1 distribution and Manders' overlap coefficients to quantify colocalization with propidium iodide nuclear staining (Table S1, NFAT localization).

Western blot analysis of the nuclear fractions of WT ESCs throughout the timeline of differentiation demonstrates that NFATC1 is detected by day 6 and peaks by days 8–12 of differentiation, after which nuclear levels sharply decline (Fig. 3, *B* and *C*). Calreticulin protein expression increases and peaks by day 6 in WT cells (Fig. 3*B*), and hence its expression pattern overlaps with NFATC1 nuclear entry within the first week of osteoblast specification. In contrast, NFATC1 nuclear entry is delayed and significantly reduced in the nonosteoblastic but chondrogenic *crt*^−/−^ cells (Fig. 3, *B* and *C*). Immunolocalization experiments throughout timeline of differentiation (Fig. 3*E*) corroborate NFATC1 cytosolic-nuclear trafficking cited in the above nuclear fractionation analysis. As a control, we measured expression of the direct target of NFATC1, *Dscr1* (21), and found that its mRNA expression profile correlated with the timing of NFATC1 nuclear translocation and that the absence of calreticulin attenuated *Dscr1* expression (Fig. 3*D*), indicating NFATC1 transcriptional activity.

Next, we inhibited intranuclear translocation of NFATC1 using a small molecule, A-285222, which inhibited intranuclear translocation of NFATC1 (Fig. S4*A*) and attenuated NFATC1 target gene, *Dscr1*, expression (Fig. 3*D*), while affecting neither NFAT phosphorylation status nor calcineurin activity (22). The absence of NFATC1 from the nucleus resulted in decreased mineralization in both differentiating WT control and *crt*^−/−^+CaN* ESCs (Fig. 2, *A* and *E*) and decreased mRNA levels of osteoblast genes *Runx2*, osterix, and osteocalcin (Fig. S4*B*), highlighting NFATs role in regulating osteoblast differentiation.

### NFATC1 suppresses chondrocyte specification

**Figure 4.**
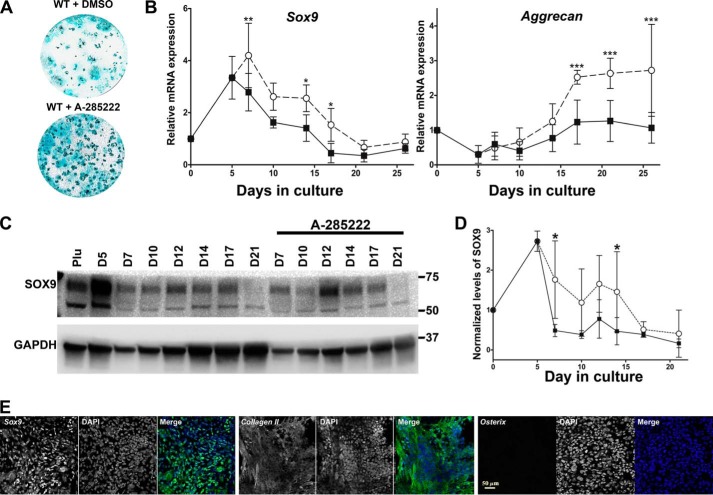
**NFAT nuclear entry determines lineage fate.** *A*, day 21 Alcian blue staining of WT ESCs treated with DMSO vehicle control or A-285222 for days 6–17 of osteoblast differentiation. *B*, RT-qPCR analysis of chondrocyte gene markers through time of differentiation; DMSO-treated WT (■) or WT ESCs treated with A-285222 (○) for days 6–17. *C* and *D*, Western blot analysis of whole-cell lysates extracted through time of differentiation (DMSO-treated WT ESCs (■); A-285222-treated WT ESCs (○)). Utilized antibodies were specific for SOX9 and internal loading standard, GAPDH. Data are expressed as means ± S.D. (*error bars*), *n* ≥ 3; two-way ANOVA. *B*, *Sox9*, *p* < 0.0001, *F* = 24.73; aggrecan, *p* < 0.0001, *F* = 42.03; *D*, *p* = 0.0001, *F* = 17.94. Bonferroni post hoc analysis was as indicated: *, *p* < 0.05; **, *p* < 0.01; ***, *p* < 0.001. *C*, confocal images of immunofluorescence localization analysis using SOX9, type II collagen, or osterix antibodies on WT ESCs at day 19 of the osteogenic differentiation protocol in the presence of A-285222 for days 6–17. Dual-channel *grayscale images* of a single field are displayed with the labeled protein of interest in the *left panel* and DAPI-stained nuclei in the *right panel*, and the *RGB panel* is a *merged image* of *green* (protein of interest) and *blue* (DAPI). See Fig. 1*E* for the WT ESC nontreated control. *Scale bar*, 50 μm.

The decrease of NFATC1 nuclear translocation in differentiating *crt*^−/−^ ESCs (Fig. 3) led us to examine whether or not NFAT regulates chondrocyte specification in cell culture favoring osteoblasts. Given that NFATC1 localizes to the nucleus around day 6 and is still present at day 17 of differentiation but at decreasing levels, we treated differentiating WT ESCs with the inhibitor of intranuclear NFAT transport, A-285222, between days 6 and 17. Preventing intranuclear presence of NFAT resulted in increased staining for chondrocyte extracellular glycosaminoglycans (Fig. 4*A*) and increased expression and stabilization of chondrocyte-specific aggrecan and chondrocyte master transcription factor *Sox9* (Fig. 4*B*) and SOX9 protein (Fig. 4, *C* and *D*). Immunofluorescence analysis at day 19 shows enhanced nuclear SOX9 and cartilage marker, type II collagen expression (Fig. 4*E*; compare with Fig. 1*E* for WT control), in the presence of A-285222. In contrast, nuclear osterix levels decreased (Fig. 4*E*). Additionally, in the presence of A-285222, expression levels of osteogenic Wnt-signaling genes *Wnt10b* and *Wnt11* were attenuated (Fig. S1) as were the osteogenic genes cited above (Fig. S4*B*).

### Calreticulin and NFAT enable GSK3β deactivation

**Figure 5.**
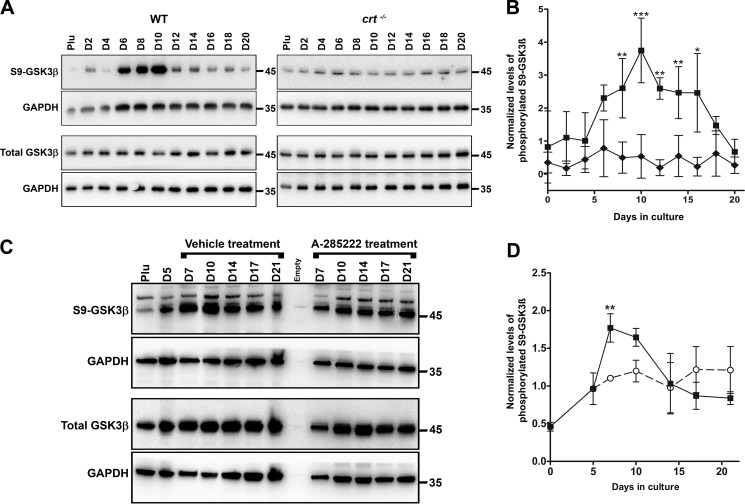
**Calreticulin and NFAT regulate GSK3β-deactivation.** *A* and *C*, Western blot analysis of whole-cell lysates harvested at the indicated times from WT control, *crt*^−/−^ ESCs (A), or WT ESCs treated with A-285222 for days 6–17 undergoing osteoblast differentiation (*C*). Utilized antibodies were specific to GSK3β, serine 9–phosphorylated GSK3β, and GAPDH as an internal control. *B* and *D*, quantitative representation of the density of the Western blotting bands of phosphorylated GSK3β normalized to internal control GAPDH. Data are expressed as means ± S.D. (*error bars*), *n* ≥ 3. Shown are WT (■), *crt*^−/−^ (♦) ESCs, and WT ESCs treated with A-285222 (○); two-way ANOVA. *B*, *p* < 0.0001, *F* = 80.51. *D*, *p* = 0.018, *F* = 6.448. Bonferroni post hoc analysis was as indicated: *, *p* < 0.05; **, *p* < 0.01; ***, *p* < 0.001.

Because we demonstrated that genes of the canonical Wnt signaling pathway are affected by calreticulin, and inhibition of GSK3β increases mice bone formation and bone mass (23, 24), we looked at the role GSK3β deactivation plays in osteoblast specification from ESCs. Using an antibody specific for the phosphorylated serine 9 of GSK3β, which marks kinase deactivation (25), Western blot analysis demonstrated that GSK3β is deactivated in a biphasic pattern overlapping with that seen in NFATC1 nucleocytoplasmic trafficking during the differentiation as demonstrated biochemically (compare Fig. 5 (*A* and *B*) with Fig. 3 (*B* and *C*)) and morphologically (Fig. 3*E*). Similar to the observation that nuclear import of NFATC1 depended on the presence of calreticulin, we show that the transient deactivation of GSK3β was also dependent on the presence of calreticulin as it was entirely absent from *crt*^−/−^ ESCs (Fig. 5, *A* and *B*). Accordingly, Ser-9 phosphorylation of GSK3β is attenuated when differentiating cultures are treated with the NFAT transport blocker A-285222 for days 6–17 (Fig. 5, *C* and *D*).

**Figure 6.**
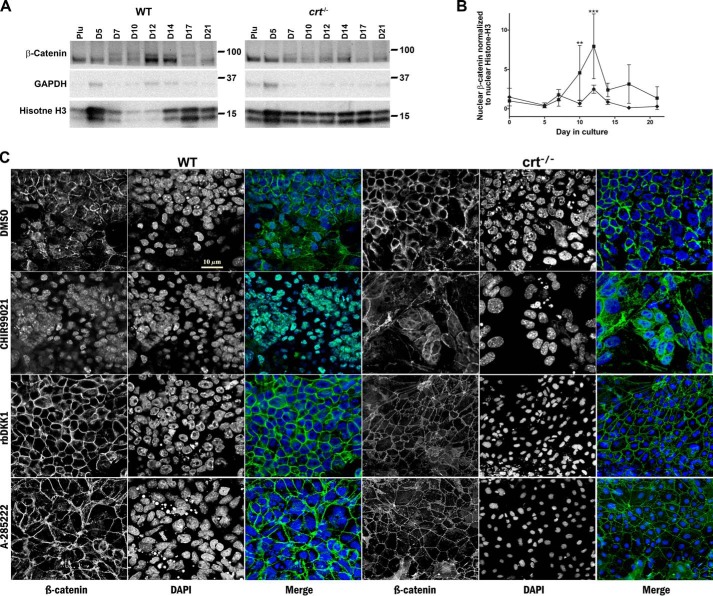
**Calreticulin regulates levels of nuclear β-catenin during osteoblast differentiation.** *A*, Western blot analysis of nuclear fractions extracted from WT control and *crt*^−/−^ ESCs on the indicated days. *B*, quantitative representation of the density of the Western blotting bands (WT control (■) and *crt*^−/−^ ESCs (♦)). Utilized antibodies were specific to β-catenin and the internal nuclear and cytosolic standards, histone H3 and GAPDH, respectively. Data are expressed as means ± S.D. (*error bars*), *n* ≥ 3; two-way ANOVA of band densities: *B*, *p* < 0.0001 and *F* = 19.76. Bonferroni post hoc test was as indicated: **, *p* < 0.01; ***, *p* < 0.001. *C*, confocal immunofluorescence images of localization of β-catenin at day 14 of the osteogenic differentiation protocol of WT control ESCs or *crt*^−/−^ ESCs with or without treatment starting at day 6 by the molecules *labeled* on the *left*. Dual-channel *greyscale images* of a single field are displayed with β-catenin in the *left panel*, DAPI-stained nuclei in the *right panel*, and the *RGB panel* of a *merged image* of *green* (β-catenin) and *blue* (DAPI). *Scale bar*, 10 μm.

A downstream consequence of GSK3β deactivation is the intranuclear accumulation of β-catenin where it induces gene transduction critical to osteoblast differentiation, while suppressing chondrocyte differentiation (16, 26). Nuclear β-catenin levels analyzed by Western blotting throughout differentiation increase in parallel to the levels of both nuclear NFATC1 and phosphorylated serine 9 GSK3β (compare Fig. 6 (*A* and *B*) with Figs. 3 (*B* and *C*) and 5 (*A* and *B*)). In contrast, nuclear β-catenin levels in *crt*^−/−^ ESCs do not significantly increase (Fig. 6, *A* and *B*). Immunolocalization of β-catenin in WT control cultures on day 14 of differentiation shows a diverse distribution of the protein that includes localization to cytosol, proximity to the plasma membrane in a pattern similar to adherens junctions, and intranuclear residence (Fig. 6*C*). In *crt*^−/−^ ESCs, β-catenin is predominately junctional and absent from nuclei (Fig. 6*C*). To assess whether β-catenin responds as anticipated in our differentiation model, we directly inhibited GSK3β with the small molecule CHIR99021 in differentiating WT ESCs. Indeed, the images demonstrate that CHIR99021 treatment restricted the entire pool of detectable cellular β-catenin to the nucleus (Fig. 6*C*). Alternatively, we treated WT cells with the canonical Wnt pathway antagonist, mouse recombinant DKK1 (rbDKK1), and that blocked intranuclear translocation of β-catenin, rendering it predominantly junctional (Fig. 6*C*). In the absence of calreticulin, or by blocking translocation of NFATC1 from the cytoplasm to the nucleus with A-285222 in both cell lines, β-catenin primarily resides in a junctional pattern (Fig. 6*C*). These results strongly suggest that NFATC1 is a link between calreticulin and GSK3β deactivation and consequently β-catenin accumulates in the nucleus, which is critical to osteoblast specification.

### Blocking canonical Wnt pathway signaling increases chondrogenesis and decreases osteogenesis

**Figure 7.**
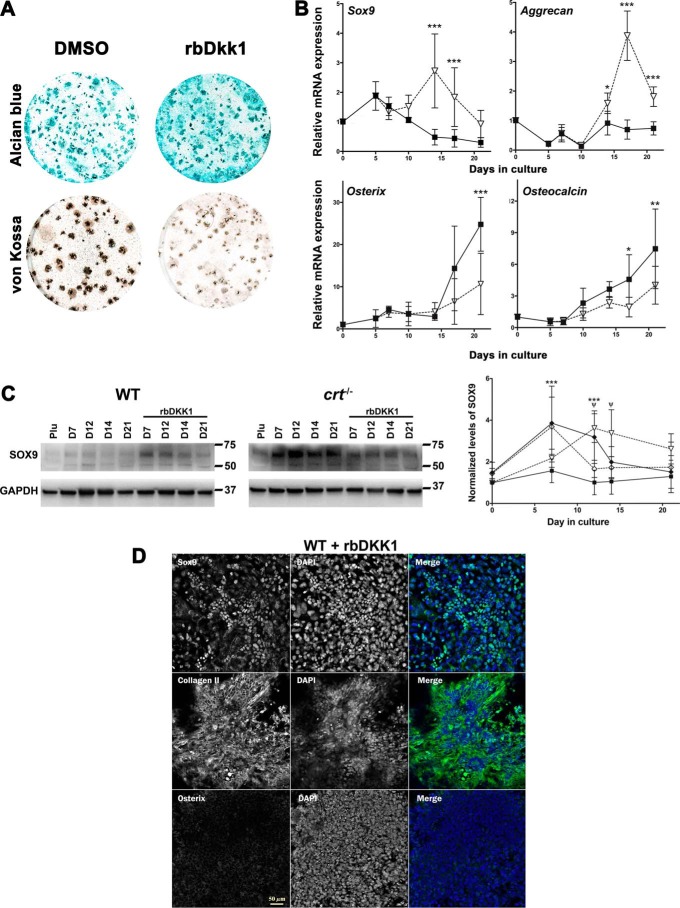
**Recombinant DKK1 increases chondrocyte specification while blocking osteoblast specification.** Differentiated WT ESCs were stained with either Alcian blue or von Kossa as indicated (*A*), treated without or with rbDKK1 for days 6–17 of osteoblast differentiation. *B*, RT-qPCR analysis of chondrocyte or osteoblast markers in WT ESCs differentiated in the absence (■) or presence (▿) of rbDKK1 for days 6–17. *C*, Western blot analysis of whole-cell lysates harvested at the indicated times from ESCs during osteoblast differentiation in the absence (■, WT ESCs; ♦, *crt*^−/−^ ESCs) or presence of mouse rbDKK1 for days 6–17 (▿, WT ESCs; ♢, *crt*^−/−^ ESCs). Utilized antibodies were specific to SOX9 and GAPDH as an internal control. The graph is a quantitative representation of the density of the Western blotting bands of SOX9 normalized to internal control GAPDH. Data are expressed as means ± S.D. (*error bars*), *n* ≥ 3; two-way ANOVA of transcript levels (*B*) and band densities (*C*). *B*, *Sox9*, *p* < 0.0001, *F* = 31.25; aggrecan, *p* < 0.0001, *F* = 94.4; osterix, *p* = 0.0073, *F* = 7.874; osteocalcin, *p* = 0.0007, *F* = 13.24. *C*, *p* < 0.0001 and *F* = 24.55 for WT control and *crt*^−/−^ ESCs data set; *p* < 0.0001 and *F* = 48.83 for WT control ESCs treated or not with rbDKK1; *p* = 0.3412 and *F* = 0.9386 for *crt*^−/−^ ESCs treated or not with rbDKK1. Bonferroni post hoc test was as indicated: *, *p* < 0.05; **, *p* < 0.01; ***, *p* < 0.001; ψ, *p* < 0.001 for WT control ESCs treated or not with rbDKK1; ***, *p* < 0.001 for WT control and *crt*^−/−^ ESCs (*C*). *D*, confocal images of immunolocalization using SOX9, type II collagen, or osterix antibodies on WT ESCs at day 19 of the osteogenic differentiation protocol in the presence of rbDKK1 on days 6–17. Dual-channel *grayscale images* of a single field are displayed with the *labeled* protein of interest in the *left panel* and DAPI-stained nuclei in the *right panel*, and the *RGB panel* is a *merged image* of *green* (protein of interest) and *blue* (DAPI). See Fig. 1*E* for the WT ESC nontreated control. *Scale bar*, 50 μm.

Because calreticulin- and NFAT-dependent deactivation of GSK3β is important for osteogenic differentiation, we hypothesized that blocking GSK3β deactivation would enhance chondrocyte differentiation during osteogenic differentiation. This is predicated by our observation that the expression of the canonical Wnt pathway inhibitor gene *Dkk1* is enhanced in chondrogenic *crt*^−/−^ ESCs or WT ESCs treated with the NFAT transport blocker A-285222 during differentiation (Fig. S1). Hence, to alleviate canonical Wnt pathway–induced GSK3β deactivation, we treated differentiating WT ESCs with recombinant mouse DKK1 on days 6–17. As expected, Western blot analysis of whole-cell lysates extracted throughout differentiation showed that rbDKK1 treatment of WT ESCs decreased levels of phosphorylated Ser-9 GSK3β (Fig. S5, *A* and *B*). This indicates that rbDKK1 blocked the canonical Wnt signaling pathway. Treatment with rbDKK1 of *crt*^−/−^ ESCs did not affect their already low levels of phosphorylation of Ser-9 GSK3β (Fig. S5, *A* and *B*). Recombinant DKK1 treatment enhanced Alcian blue staining (Fig. 7*A*), increased transcript expression of chondrocyte marker genes *Sox9* and aggrecan (Fig. 7*B*), and increased levels of SOX9 protein expression, but did not significantly change expression levels of SOX9 in *crt*^−/−^ cells (Fig. 7*C*). There was also a concomitant decrease of mineralization (Fig. 7*A*) and osteoblast gene expression of osterix and osteocalcin (Fig. 7*B*). Immunofluorescence analysis shows that rbDKK1 treatment increased SOX9 nuclear localization and type II collagen staining and decreased osterix nuclear localization (Fig. 7*D*). Recombinant DKK1 induced an increased junctional-like localization of β-catenin in WT cells first cited above (Fig. 6*C*). This observation corroborates with the Western blot analysis of WT nuclear fractions that shows that rbDKK1 treatment decreased nuclear β-catenin levels compared with nontreated cells and that in the nuclear fractions of differentiating *crt*^−/−^ ESCs the lower levels of β-catenin do not significantly change regardless of rbDKK1 treatment (Fig. S5, *C* and *D*). These results confirm that osteogenic ESC differentiation depends on the canonical Wnt signaling pathway and that disruption of calreticulin, NFAT nuclear translocation, or canonical Wnt signaling induces chondrocyte specification in conditions that otherwise favor the differentiation of ESCs to osteoblasts.

We next considered whether the disruption of canonical Wnt signaling by rbDKK1 alters NFATC1 translocation to the nucleus and thereby negatively affects this pro-osteogenic event. Western blot analysis of WT and *crt*^−/−^ ESC nuclear fractions showed that rbDKK1 did not significantly alter the levels of nuclear NFATC1 in the differentiating WT nuclear fractions nor the blockage of NFATC1 nuclear transport observed in nontreated *crt*^−/−^ ESC nuclear fractions (Fig. S5, *E* and *F*). This observation supports the role for nuclear NFAT transport—dependent on calreticulin—to positively regulate a pro-osteoblast differentiation response by the canonical Wnt signaling pathway that concomitantly suppresses chondrocyte differentiation from a culture of ESCs.

### Calreticulin via GSK3β deactivation enhances osteoblast specification as it suppresses chondrocyte specification

**Figure 8.**
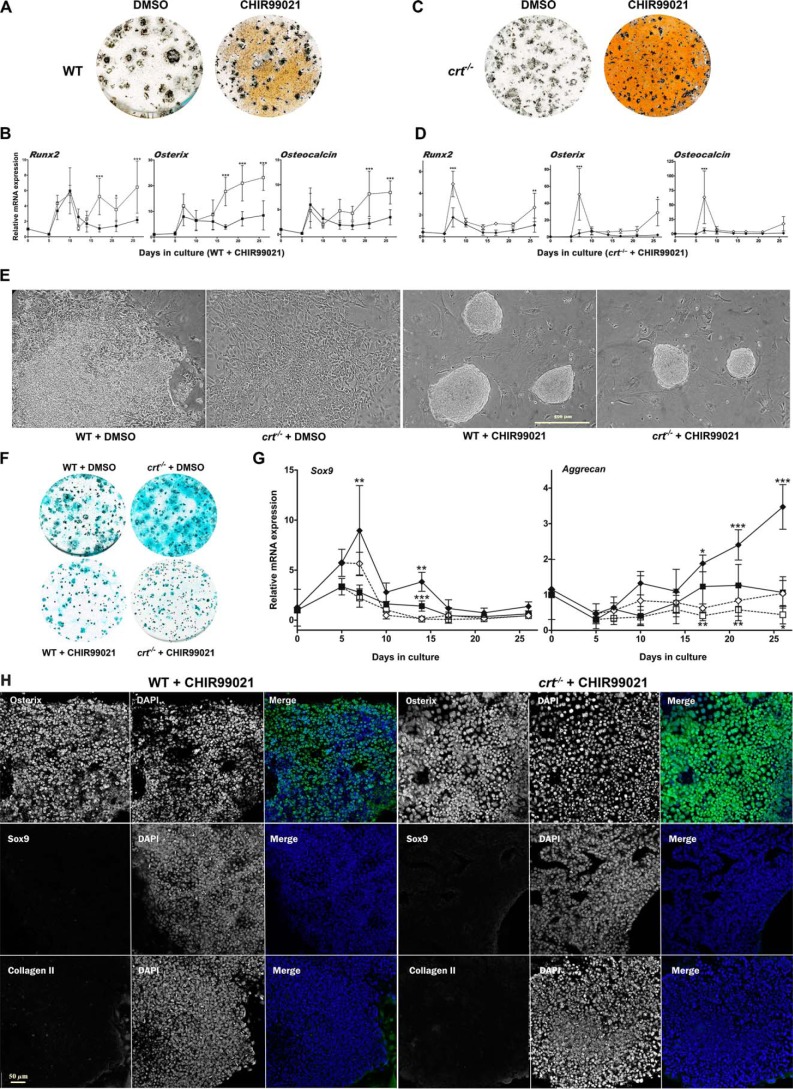
**GSK3β deactivation promotes osteoblast specification and suppresses chondrocyte specification from ESCs.** *A–D*, day 26 von Kossa staining of WT ESCs (*A*) or *crt*^−/−^ ESCs (*C*) and RT-qPCR analysis of osteoblast genes in WT ESCs (*B*) treated with DMSO vehicle control (■) or CHIR99021 (□) for days 6–17 or *crt*^−/−^ ESCs (*D*) treated with DMSO vehicle control (♦) or CHIR99021 (♢) for days 6–17 of osteoblast differentiation. *E*, phase-contrast images of day 10 differentiated WT cells or *crt*^−/−^ cells treated with vehicle control, DMSO, or CHIR99021 for days 6–10. *Scale bar*, 500 μm. *F*, day 21 Alcian blue staining for WT and *crt*^−/−^ cells treated with DMSO or CHIR99021 for days 6–17. *G*, RT-qPCR analysis of chondrocyte markers through time. ESCs were treated with DMSO (WT ESCs (■); *crt*^−/−^ ESCs (♦)) or CHIR99021 (WT ESCs (□); *crt*^−/−^ ESCs (♢)) for days 6–17 of differentiation. Data are expressed as means ± S.D. (*error bars*), *n* ≥ 3. *H*, confocal immunofluorescence localization of osterix, SOX9, or type II collagen at day 19 of the osteogenic differentiation protocol of WT ESCs or *crt*^−/−^ ESCs treated with CHIR99021 for days 6–17 of the osteoblast differentiation protocol. Dual-channel *grayscale* images of a single field are displayed with the protein of interest *labeled* in the *left panel* and DAPI-stained nuclei in the *right panel*. The *RGB panel* shows a *merged image* of *green* (protein of interest) and *blue* (DAPI). See Fig. 1*E* for the respective nontreated ESC line control. *Scale bar*, 50 μm; two-way ANOVA. *B*, *Runx2*, *p* < 0.0001, *F* = 20.53; osterix, *p* < 0.0001, *F* = 52.42; osteocalcin, *p* < 0.0001, *F* = 20.05. *D*, *Runx2*, *p* < 0.0001, *F* = 29.55; osterix, *p* = 0.0001, *F* = 18.71; osteocalcin, *p* = 0.0023, *F* = 10.84. *G*, transcript levels between WT control and *crt*^−/−^ ESCs (*Sox9*, *p* < 0.0001, *F* = 41.62; aggrecan, *p* < 0.0001, *F* = 53.95); transcript levels between WT control ESCs treated or not with CHIR99021 (*Sox9*, *p* = 0.0001, *F* = 16.89; aggrecan, *p* < 0.0001, *F* = 19.62); transcript levels between *crt*^−/−^ ESCs treated or not with CHIR99021 (*Sox9*, *p* < 0.0001, *F* = 20.52; aggrecan, *p* < 0.0001, *F* = 39.2). Bonferroni post hoc analysis was as indicated (*B*, *D*, and *G*): *, *p* < 0.05; **, *p* < 0.01; ***, *p* < 0.001.

To examine the effect of GSK3β deactivation in differentiation, we enhanced and prolonged the GSK deactivation of the differentiating WT ESC population with the potent and specific inhibitor, CHIR99021 between days 6 and 17. There was a significant increase of mineralization as visualized by von Kossa staining (Fig. 8*A*) and enhanced expression of the osteoblast markers *Runx2*, osterix, and osteocalcin (Fig. 8*B*). In the presence of CHIR99021, the WT cells grow into tightly packed spherical nodules by day 10 (Fig. 8*E*). Immunofluorescence analysis of these packed nodules at day 19 shows substantial levels of nuclear osterix (Fig. 8*H*) and enhanced nuclear β-catenin (Fig. 6*C*, *CHIR99021 row*) and low levels of both nuclear SOX9 and extracellular type II collagen immunostaining (Fig. 8*H*).

We propose that direct inhibition of GSK3β would mitigate the requirement of calreticulin, calcineurin, and NFATC1, which are all absent or attenuated in differentiating *crt*^−/−^ ESCs. Indeed, inhibition of GSK3β in differentiating *crt*^−/−^ ESCs increased mineralization, comparable with control ESCs (Fig. 8*C*). Osteoblast gene expression profiles of *crt*^−/−^ ESCs in the presence of CHIR99021 are elevated (Fig. 8*D*). Phase-contrast morphology of the compact nodules grown in the presence of CHIR99021 is also indistinguishable between the WT and *crt*^−/−^ cell lines (Fig. 8*E*). In contrast, CHIR99021-induced inhibition of GSK3β decreased Alcian blue staining in both WT and *crt*^−/−^ ESCs to similar levels (Fig. 8*F*). Transcript levels of aggrecan and *Sox9* were suppressed in WT cells (Fig. 8*G*). Likewise, CHIR99021 treatment of *crt*^−/−^ ESCs suppressed the inherently enhanced expression of *Sox9* and aggrecan (Fig. 8*G*). CHIR99021 treatment of *crt*^−/−^ ESCs also increased the abundance of nuclear osterix (Fig. 8*H*) and nuclear β-catenin (Fig. 6*C*, *CHIR99021 row*), while decreasing nuclear SOX9 and expression of the cartilage marker type II collagen (Fig. 8*H*; compare with *crt*^−/−^ cells treated with vehicle control in Fig. 1*E*) similarly to WT ESCs treated with CHIR99021. We observe that CHIR99021 treatment of *crt*^−/−^ ESCs did not enhance osteogenic gene expression as robustly as CHIR99021 treatment of WT ESCs (Fig. 8, *B* and *D*). Likewise, β-catenin is not as effectively sequestrated to the nuclei of CHIR99021-treated *crt*^−/−^ ESCs (Fig. 6*C*, *CHIR99021 row*). This suggests that calreticulin has additional roles in the maintenance of osteoblast functionality, which might extend to other signaling pathways or to the stability and trafficking of β-catenin. Collectively, these data demonstrate that, in differentiating ESCs, calreticulin-dependent deactivation of GSK3β promotes osteoblast specification while at the same time suppressing chondrocyte specification.

## Discussion

We propose that calreticulin conducts a Ca^2+^-mediated signal from the ER to promote osteoblast specification from ESCs. The Ca^2+^-buffering capacity of calreticulin is chiefly responsible for the induction of the osteoblast program, which is mediated by the cytosolic Ca^2+^-dependent phosphatase, calcineurin. Calcineurin dephosphorylates and induces the nuclear translocation of NFATC1 as part of the signaling pathway for bone formation (5, 10, 11). Concurrently, calreticulin also suppresses chondrocyte specification from differentiating ESCs. We demonstrate for the first time that blocking NFAT nuclear transport, either by calreticulin ablation or use of a specific NFAT inhibitor, enhanced and stabilized the expression of *Sox9*. Consequently, by controlling the locus of NFATC1 residence, calreticulin regulates a molecular switch in the fate of differentiating ESCs to osteoblasts or chondrocytes.

Another important signaling event necessary for bone formation is the deactivation of GSK3β (23, 24). Deactivation of GSK3β in WT cells during osteoblast differentiation overlaps with NFATC1 residence in the nucleus. In the absence of calreticulin, GSK3β deactivation is abolished. This strongly implies that calreticulin affects the osteogenic signaling mechanisms that deactivate GSK3β. We found that blocking NFAT nuclear translocation also blocked deactivation of GSK3β, possibly through NFAT-dependent stimulation of canonical Wnt pathway genes, such as *Wnt10b* (14), and suppression of the Wnt signaling antagonist *Dkk1* (15). Indeed, we found that during differentiation, *Wnt10b* expression depended on calreticulin and NFAT signaling and that at similar differentiation periods, *crt*^−/−^ cell cultures had enhanced *Dkk1* expression, which was also enhanced in WT ESCs upon treatment with the NFAT blocker A-285222 (Fig. S1). DKK1 is closely associated with lytic bone lesions in patients with multiple myeloma (27), and neutralizing secreted DKK1 protected bone from damage in arthritic joints (28). These cited studies indicate that DKK1 impedes bone integrity by disrupting osteoblast differentiation and are supported by our observations that treatment of WT ESCs with recombinant DKK1 also disrupted osteoblast differentiation. Instead, recombinant DKK1 stimulated the expression of chondrocyte characteristics supporting previous studies demonstrating that DKK1 was indispensable for the promotion of chondrogenic differentiation of human mesenchymal stem cells (29).

The situation whereby calreticulin stimulates canonical Wnt pathway signaling and suppresses its antagonism augments deactivation of GSK3β for osteoblastogenesis at the expense of chondrocyte formation. To further explore this, we inhibited GSK3β with CHIR99021, which rescued osteoblast output in otherwise chondrogenic *crt*^−/−^ ESCs to levels like those in control WT ESCs treated for GSK3β inhibition. CHIR99021 treatment also robustly increased functional osteoblast output compared with the previously published differentiation protocol that relies on high serum levels and yields heterogeneity (30). The ability of CHIR99021 to alter the outcome of differentiation indicates that within a week of the start of differentiation, the cell population is amenable to either osteoblast or chondrocyte specification. Indeed, GSK3β inhibition starting on day 6 suppressed the expression of *Sox9* in both cell lines. *Sox9* is a potent and dominant inhibitor of *Runx2* (31) and promotes chondrogenesis. Conversely, stabilized canonical Wnt/β-catenin signaling reduces the expression of *Sox9* (16) while promoting skeletogenesis. Kumar and Lassar (32) irreversibly silenced the *Sox9* gene by canonical Wnt signaling–induced activation of the DNA methyltransferase, DNMT3A. This agrees with our data, which demonstrate that pharmacologic inhibition of GSK3β significantly suppresses *Sox9* expression, ensuring that a higher level of *Sox9* does not interfere with specification of osteoblasts.

Anatomically (33, 34) and functionally (1), the ER is a signaling organelle that plays an important role in development (3). Calreticulin, acting as a Ca^2+^-sensitive spigot for ER Ca^2+^ stores, affects outcomes of developmental decisions by controlling trafficking of transcriptionally crucial factors. Little is known about what directly controls calreticulin function during cell lineage specification, but its effect on the locus of NFATC1 residence and the status of GSK3β activation suggests a mechanism that asserts the essential mutual exclusivity of the osteoblast and chondrocyte lineages in skeletal development.

## Materials and methods

### Cell culture and ESC differentiation

G45 calreticulin knockout (*crt*^−/−^) mouse ESCs (7), WT ESCs (R1, derived from J1 129/Sv mice, purchased from the Embryonic Stem Cell Facility, Laboratory of Dr. Janet Rossant, Developmental and Stem Cell Biology, Hospital for Sick Children, Toronto, Canada) (35), and *crt*^−/−^ cells stably transfected with constitutively active calcineurin (*crt*^−/−^+CaN* (19)) were maintained on feeder cells of mitomycin C (Sigma)-treated mitotically inactive mouse embryonic fibroblasts on gelatin-coated plates. The ESCs were cultured in high-glucose ESC-optimized Dulbecco's modified Eagle's medium (Multicell, Wisent), supplemented with 10% premium fetal bovine serum (virus- and mycoplasma-screened; Multicell, Wisent), 10 ng/ml recombinant leukemia-inhibitory factor, nonessential amino acids (Gibco), and 0.1 mm β-mercaptoethanol (BioShop). To initiate differentiation, we followed the protocol described by Yu *et al.* (36), but in brief, ESCs were trypsinized to single cells and suspended in base differentiation medium (high-glucose Dulbecco's modified Eagle's medium supplemented in 20% premium fetal bovine serum, nonessential amino acid solution, β-mercaptoethanol). On average, 250 cells were allocated per 25-μl drop and hung upside-down on a Petri dish lid for 3 days to generate spherical differentiated cell aggregates called embryoid bodies. They were collected in base differentiation medium supplemented with 0.1 μm retinoic acid (Sigma) and into Petri dishes that did not permit embryoid body (EB) surface attachment. After 2 days (day 5), the EBs were directly plated onto gelatin-coated tissue culture–treated plates, or to reduce cardiomyocyte formation, we trypsinized the EBs, filtered the suspension for single cells through a 100-μm nylon cell strainer (BD Falcon), and plated on tissue culture–treated plates at a density of 30,000 cells/cm^2^ in differentiation medium supplemented with 50 μg/ml ascorbic acid (Sigma) and 10 mm β-glycerophosphate (Sigma) for specification and maintenance of the osteoblast lineage. On day 10, dexamethasone (Sigma) at 100 nm was added, and medium supplemented with ascorbic acid, β-glycerophosphate, and dexamethasone was changed every other day for 21 or 26 days.

### Ca^2+^ manipulation and inhibitor studies

Differentiating cultures were treated with 0.1 μm cell membrane–permeable Ca^2+^ chelator BAPTA-AM (Sigma) for a 30-min incubation on each of days 3–5 of differentiation. Differentiating cultures were treated with the Ca^2+^ ionophore ionomycin (Sigma) at 1 μm for 2 h on each of days 3–5. For inhibition of calcineurin at days 3–5, cultures were treated with 1 μg/ml cyclosporin A (Sigma) for 2 h each day. Differentiating cells were treated with the inhibitor of intranuclear transport of NFAT A-285222 (*M*_r_ 416.28) (22) (a generous gift from AbbVie) at 5 μg/ml, a 3 μm concentration of the GSK inhibitor CHIR99021 (Tocris), or 100 ng/ml mouse recombinant DKK1 (R&D Systems), each continuously for days 6–17.

### Calcineurin activity assay

Calcineurin activity was assessed as described previously by Fruman *et al.* (37). Briefly, a peptide corresponding to the regulatory domain of protein kinase A (RII-peptide, Sigma) was used as the substrate in an *in vitro* dephosphorylation assay. 1.0 × 10^6^ ESCs were lysed in hypotonic buffer (50 mm Tris, pH 7.5, 0.1 mm EGTA, 1 mm EDTA, 250 mm DTT, and protease inhibitors). γ-^32^P-radiolabeled RII-peptide was added to a fraction of the lysate, and the dephosphorylation reaction was carried out for 20 min at 30 °C in the presence of 0.5 mm okadaic acid to eliminate contaminating serine/threonine phosphatases. Released radiolabeled phosphate was isolated away from the RII-peptide, and calcineurin phosphatase activity was expressed in pmol of phosphate released per mg of total protein.

### Mineral deposition assays and Alcian blue staining

Differentiated cell cultures were stained for the presence of mineral deposits using von Kossa and alizarin red S (Sigma) as described in previous studies (38). Briefly, for von Kossa staining, cells were rinsed three times with PBS, fixed in 10% neutral formalin buffer for 2 h, and then washed three times with distilled water. The fixed cells were stained with 2.5% silver nitrate solution for 30 min in the presence of light produced from a 100-watt incandescent light bulb and then washed three times with distilled water. The amount of Ca^2+^ in the matrix was measured using the Arsenazo III (Sigma) staining method described previously (39). To visualize acidic proteoglycans secreted by chondrocytes, we stained 10% formalin-fixed cells with 1% Alcian blue 8GX (Sigma; 1 g of Alcian blue 8GX dissolved in 100 ml of 0.1 n HCl) for 30 min and then washed twice with 0.1 n HCl. All of the fixed and stained cells were air-dried, and the images were captured using an Epson Perfection V750 flat-bed light scanner.

### Reverse transcriptase real-time PCR analysis

The Macherey–Nagel NucleoSpin RNA plus kit (D-Mark Biosciences) was used to extract total RNA from ESCs and differentiating cell progeny. 1 μg of total RNA was reverse-transcribed to cDNA using Bio-Rad iScript cDNA synthesis kit. Real-time PCR analysis was performed in Bio-Rad's CFX384 Touch detection system. The accumulation of PCR products was monitored by measuring the fluorescence produced by the binding of SYBR Green from the SensiFAST SYBR No-ROX kit (Bioline) to dsDNA. The cDNA levels were normalized with *L32* (a housekeeping gene). Primer sequences are listed in Table S2.

### SDS-PAGE and Western blot analysis

Cell lysates were collected in buffer that contained 50 mm Tris-HCl, pH 8.0, 120 mm NaCl, and 0.5% Nonidet P-40. The Bradford method was used to quantify the concentrations of protein samples (40). Protein samples (10–30 μg/lane) were separated by SDS-PAGE (7.5% or 4–20% gradient gels; Bio-Rad) and transferred to nitrocellulose membrane. Rabbit anti-SOX9 (EMD Millipore) and mouse anti-b-catenin (BD Transduction Laboratories) were used at a 1:2000 dilution. The following primary antibodies were used at 1:1000 dilution (Cell Signaling Technology): anti-GSK3β, anti-phospho-GSK3β (Ser-9), anti-glyceraldehyde-3-phosphate dehydrogenase (GAPDH), anti-NFAT2, anti-histone H3. The secondary antibody used was goat polyclonal secondary antibody to rabbit IgG-H&L (horseradish peroxidase) (Abcam). Immunoreactive bands were detected using the DNR MicroChem chemiluminescence camera system (DNR Bio-Imaging Systems). Bands were quantified with GelQuant software.

### Immunofluorescence staining

Cells plated on gelatin-coated coverslips were rinsed three times with PBS and then fixed in 3.7% formaldehyde for 15 min. After washing (three times for 5 min each time) in PBS, the cells were permeabilized with 0.1% Triton X-100 in buffer containing 100 mm PIPES, 1 mm EGTA, and 4% (w/v) PEG 8000 (pH 6.9) for 15 min. Fixed cells were then washed three times in PBS for 5 min and incubated with rabbit anti-NFAT2 (Cell Signaling Technology; dilution of 1:50), rabbit anti-SOX9 (EMD Millipore; 1:500), rabbit anti-Sp7/osterix and rabbit anti-collagen II (Abcam; 1:250), and mouse anti-β-catenin (BD Transduction Laboratories; 1:500) overnight at 4 °C. After washing in PBS (three times for 5 min), the cells were stained with the secondary antibody (FITC-conjugated donkey anti-rabbit (1:50) or Alexa Fluor 594 goat anti-rabbit (1:1000)) for 1 h at room temperature. The cells were washed three times in PBS for 5 min and incubated with 100 μg/ml DNase-free RNase in PBS for 30 min at 37 °C, followed by rinsing in PBS three times for 1 min each. For nuclear staining, 500 nm propidium iodide (PI) solution was added to cover the cells for 5 min. After a final rinse in PBS, the slides were mounted in fluorescent mounting medium (Dako) or ProLong Gold Antifade with DAPI (Molecular Probes). To confirm the specificities of the secondary antibodies used, we performed immunofluorescence staining using calreticulin WT cells omitting primary antibodies. There was no nonspecific binding seen with anti-rabbit secondary antibodies. A confocal fluorescence microscope (MRC 600, Bio-Rad) equipped with a ×60/1.40 numerical aperture plan apochromatic oil immersion objective (Nikon) and krypton-argon laser was used for fluorescence imaging. COMOS software (Bio-Rad) was used for image acquisition. Additionally, we used an LSM 880 Elyra super-resolution confocal microscope with a ×20/0.8 numerical aperture M27 plan apochromatic objective and ZEN blue edition (Carl Zeiss) for image processing. Mander's coefficients were calculated using ImageJ (National Institutes of Health).

### Subcellular fractionation

Nuclear fractions were obtained by a modification of the method described previously (41). Briefly, cells were collected and washed with ice-cold 1× PBS, lysed on ice for 30 min in cold cytosolic extraction buffer (20 mm sucrose, 50 mm Tris-HCl, pH 7.4, 5 mm MgCl_2_, and protease and phosphatase inhibitors). The cytosolic protein was collected by centrifugation at 800 × *g* for 15 min at 4 °C. The pelleted nuclei was treated with nuclear extraction buffer (20 mm HEPES, pH 7.9, 1.5 mm MgCl_2_, 0.5 mm EDTA, 20% glycerol, 1% Triton X-100, and protease and phosphatase inhibitors). The nuclei were incubated with the nuclear extraction buffer on ice for 30 min followed by centrifugation at 9000 × *g* for 30 min at 4 °C to remove cellular debris from the nuclear extract. Protein concentration was measured for both the nuclear and cytosolic fractions using the Bio-Rad DC Protein Assay followed by denaturing and boiling with 2× SDS-PAGE sample buffer.

### Statistical analysis

Statistical differences were calculated by one- or two-way analysis of variance followed by a Bonferroni post hoc test using GraphPad Prism 3.02.

### Data availability

All data are contained within the article.

## Author contributions

C. P. and M. O. conceptualization; C. P. and Z. A. data curation; C. P. and Z. A. formal analysis; C. P. and M. O. supervision; C. P. and M. O. investigation; C. P., Z. A., and M. O. visualization; C. P., Z. A., and M. O. methodology; C. P. and M. O. writing-original draft; C. P., Z. A., and M. O. writing-review and editing; Z. A. validation; M. O. funding acquisition; M. O. project administration.

## Supplementary Material

Supporting Information

## Footnotes

3 The abbreviations used are:

ER: endoplasmic reticulum
ESC: embryonic stem cell
NFAT: nuclear factor of activated T cells
NFATC: cytoplasmic NFAT
rbDKK1: recombinant DKK1
EB: embryoid body
GAPDH: glyceraldehyde-3-phosphate dehydrogenase
DAPI: 4′,6-diamidino-2-phenylindole
RT-qPCR: real-time quantitative RT-PCR
PI: propidium iodide
ANOVA: analysis of variance.

## References

[1] Berridge M. J. (2002) The endoplasmic reticulum: a multifunctional signalling organelle. Cell Calcium 32, 235–249 10.1016/S0143416002001823 12543086

[2] Michalak M., Parker J. M. R., and Opas M. (2002) Ca^2+^ signaling and calcium binding chaperones of the endoplasmic reticulum. Cell Calcium 32, 269–278 10.1016/S0143416002001884 12543089

[3] Webb S. E., and Miller A. L. (2003) Calcium signalling during embryonic development. Nat. Rev. Mol. Cell Biol. 4, 539–551 10.1038/nrm1149 12838337

[4] Tonelli F. M., Santos A. K., Gomes D. A., da Silva S. L., Gomes K. N., Ladeira L. O., and Resende R. R. (2012) Stem cells and calcium signalling. Adv. Exp. Med. Biol. 740, 891–916 10.1007/978-94-007-2888-2_40 22453975 PMC3979962

[5] Crabtree G. R., and Olson E. N. (2002) NFAT signalling: Choreographing the social lives of cells. Cell 109, S67–S79 10.1016/S0092-8674(02)00699-2 11983154

[6] Groenendyk J., Lynch J., and Michalak M. (2004) Calreticulin, Ca^2+^, and calcineurin: signalling from the endoplasmic reticulum. Mol. Cells 17, 383–389 15232210

[7] Mesaeli N., Nakamura K., Zvaritch E., Dickie P., Dziak E., Krause K. H., Opas M., MacLennan D. H., and Michalak M. (1999) Calreticulin is essential for cardiac development. J. Cell Biol. 144, 857–868 10.1083/jcb.144.5.857 10085286 PMC2148186

[8] Guo L., Nakamura K., Lynch J., Opas M., Olson E. N., Agellon L. B., and Michalak M. (2002) Cardiac-specific expression of calcineurin reverses embryonic lethality in calreticulin-deficient mouse. J. Biol. Chem. 277, 50776–50779 10.1074/jbc.M209900200 12377773

[9] Sun L., Blair H. C., Peng Y., Zaidi N., Adebanjo O. A., Wu X. B., Wu X. Y., Iqbal J., Epstein S., Abe E., Moonga B. S., and Zaidi M. (2005) Calcineurin regulates bone formation by the osteoblast. Proc. Natl. Acad. Sci. U.S.A. 102, 17130–17135 10.1073/pnas.0508480102 16286645 PMC1288002

[10] Koga T., Matsui Y., Asagiri M., Kodama T., de Crombrugghe B., Nakashima K., and Takayanagi H. (2005) NFAT and Osterix cooperatively regulate bone formation. Nat. Med. 11, 880–885 10.1038/nm1270 16041384

[11] Winslow M. M., Pan M., Starbuck M., Gallo E. M., Deng L., Karsenty G., and Crabtree G. R. (2006) Calcineurin/NFAT signaling in osteoblasts regulates bone mass. Dev. Cell 10, 771–782 10.1016/j.devcel.2006.04.006 16740479

[12] Long F. (2011) Building strong bones: molecular regulation of the osteoblast lineage. Nat. Rev. Mol. Cell Biol. 13, 27–38 10.1038/nrm3254 22189423

[13] Craft A. M., Ahmed N., Rockel J. S., Baht G. S., Alman B. A., Kandel R. A., Grigoriadis A. E., and Keller G. M. (2013) Specification of chondrocytes and cartilage tissues from embryonic stem cells. Development 140, 2597–2610 10.1242/dev.087890 23715552

[14] Lerner U. H., and Ohlsson C. (2015) The WNT system: background and its role in bone. J. Intern. Med. 277, 630–649 10.1111/joim.12368 25845559

[15] Pinzone J. J., Hall B. M., Thudi N. K., Vonau M., Qiang Y. W., Rosol T. J., and Shaughnessy J. D. Jr. (2009) The role of Dickkopf-1 in bone development, homeostasis, and disease. Blood 113, 517–525 10.1182/blood-2008-03-145169 18687985 PMC2628360

[16] Hill T. P., Später D., Taketo M. M., Birchmeier W., and Hartmann C. (2005) Canonical Wnt/β-catenin signaling prevents osteoblasts from differentiating into chondrocytes. Dev. Cell 8, 727–738 10.1016/j.devcel.2005.02.013 15866163

[17] Michalak M., Groenendyk J., Szabo E., Gold L. I., and Opas M. (2009) Calreticulin, a multi-process calcium-buffering chaperone of the endoplasmic reticulum. Biochem. J. 417, 651–666 10.1042/BJ20081847 19133842

[18] Lynch J., and Michalak M. (2003) Calreticulin is an upstream regulator of calcineurin. Biochem. Biophys. Res. Commun. 311, 1173–1179 10.1016/j.bbrc.2003.08.040 14623303

[19] Lynch J., Guo L., Gelebart P., Chilibeck K., Xu J., Molkentin J. D., Agellon L. B., and Michalak M. (2005) Calreticulin signals upstream of calcineurin and MEF2C in a critical Ca^2+^-dependent signaling cascade. J. Cell Biol. 170, 37–47 10.1083/jcb.200412156 15998798 PMC2171392

[20] Sitara D., and Aliprantis A. O. (2010) Transcriptional regulation of bone and joint remodeling by NFAT. Immunol. Rev. 233, 286–300 10.1111/j.0105-2896.2009.00849.x 20193006 PMC2904911

[21] Fuentes J. J., Genescà L., Kingsbury T. J., Cunningham K. W., Pérez-Riba M., Estivill X., and de la Luna S. (2000) DSCR1, overexpressed in Down syndrome, is an inhibitor of calcineurin-mediated signaling pathways. Hum. Mol. Genet. 9, 1681–1690 10.1093/hmg/9.11.1681 10861295

[22] Djuric S. W., BaMaung N. Y., Basha A., Liu H., Luly J. R., Madar D. J., Sciotti R. J., Tu N. P., Wagenaar F. L., Wiedeman P. E., Zhou X., Ballaron S., Bauch J., Chen Y. W., Chiou X. G., et al (2000) 3,5-Bis(trifluoromethyl)pyrazoles: a novel class of NFAT transcription factor regulator. J. Med. Chem. 43, 2975–2981 10.1021/jm990615a 10956206

[23] Clément-Lacroix P., Ai M., Morvan F., Roman-Roman S., Vayssière B., Belleville C., Estrera K., Warman M. L., Baron R., and Rawadi G. (2005) Lrp5-independent activation of Wnt signaling by lithium chloride increases bone formation and bone mass in mice. Proc. Natl. Acad. Sci. U.S.A. 102, 17406–17411 10.1073/pnas.0505259102 16293698 PMC1297659

[24] Kugimiya F., Kawaguchi H., Ohba S., Kawamura N., Hirata M., Chikuda H., Azuma Y., Woodgett J. R., Nakamura K., and Chung U. I. (2007) GSK-3β controls osteogenesis through regulating Runx2 activity. PLoS ONE 2, e837 10.1371/journal.pone.0000837 17786208 PMC1950686

[25] Stambolic V., and Woodgett J. R. (1994) Mitogen inactivation of glycogen synthase kinase-3β in intact cells via serine 9 phosphorylation. Biochem. J. 303, 701–704 10.1042/bj3030701 7980435 PMC1137602

[26] Day T. F., Guo X., Garrett-Beal L., and Yang Y. (2005) Wnt/β-catenin signaling in mesenchymal progenitors controls osteoblast and chondrocyte differentiation during vertebrate skeletogenesis. Dev. Cell 8, 739–750 10.1016/j.devcel.2005.03.016 15866164

[27] Tian E., Zhan F., Walker R., Rasmussen E., Ma Y., Barlogie B., and Shaughnessy J. D. Jr. (2003) The role of the Wnt-signaling antagonist DKK1 in the development of osteolytic lesions in multiple myeloma. N. Engl. J. Med. 349, 2483–2494 10.1056/NEJMoa030847 14695408

[28] Diarra D., Stolina M., Polzer K., Zwerina J., Ominsky M. S., Dwyer D., Korb A., Smolen J., Hoffmann M., Scheinecker C., van der Heide D., Landewe R., Lacey D., Richards W. G., and Schett G. (2007) Dickkopf-1 is a master regulator of joint remodeling. Nat. Med. 13, 156–163 10.1038/nm1538 17237793

[29] Zhong L., Huang X., Rodrigues E. D., Leijten J. C., Verrips T., El Khattabi M., Karperien M., and Post J. N. (2016) Endogenous DKK1 and FRZB regulate chondrogenesis and hypertrophy in three-dimensional cultures of human chondrocytes and human mesenchymal stem cells. Stem Cells Dev. 25, 1808–1817 10.1089/scd.2016.0222 27733096 PMC5124737

[30] Yu Y., Al-Mansoori L., and Opas M. (2015) Optimized osteogenic differentiation protocol from R1 mouse embryonic stem cells *in vitro*. Differentiation 89, 1–10 10.1016/j.diff.2014.12.003 25613029

[31] Zhou G., Zheng Q., Engin F., Munivez E., Chen Y., Sebald E., Krakow D., and Lee B. (2006) Dominance of SOX9 function over RUNX2 during skeletogenesis. Proc. Natl. Acad. Sci. U.S.A. 103, 19004–19009 10.1073/pnas.0605170103 17142326 PMC1748167

[32] Kumar D., and Lassar A. B. (2014) Fibroblast growth factor maintains chondrogenic potential of limb bud mesenchymal cells by modulating DNMT3A recruitment. Cell Rep. 8, 1419–1431 10.1016/j.celrep.2014.07.038 25159139 PMC4163101

[33] Papp S., Dziak E., Michalak M., and Opas M. (2003) Is all of the endoplasmic reticulum created equal? The effects of the heterogeneous distribution of endoplasmic reticulum Ca^2+^-handling proteins. J. Cell Biol. 160, 475–479 10.1083/jcb.200207136 12591911 PMC2173736

[34] Schwarz D. S., and Blower M. D. (2016) The endoplasmic reticulum: structure, function and response to cellular signaling. Cell Mol. Life Sci. 73, 79–94 10.1007/s00018-015-2052-6 26433683 PMC4700099

[35] Nagy A., Rossant J., Nagy R., Abramow-Newerly W., and Roder J. C. (1993) Derivation of completely cell culture-derived mice from early-passage embryonic stem cells. Proc. Natl. Acad. Sci. U.S.A. 90, 8424–8428 10.1073/pnas.90.18.8424 8378314 PMC47369

[36] Yu Y., Pilquil C., and Opas M. (2016) Osteogenic differentiation from embryonic stem cells. Methods Mol. Biol. 1341, 425–435 10.1007/7651_2014_126 25417061

[37] Fruman D. A., Mather P. E., Burakoff S. J., and Bierer B. E. (1992) Correlation of calcineurin phosphatase activity and programmed cell death in murine T cell hybridomas. Eur. J. Immunol. 22, 2513–2517 10.1002/eji.1830221008 1382988

[38] Bellows C. G., Aubin J. E., Heersche J. N., and Antosz M. E. (1986) Mineralized bone nodules formed in vitro from enzymatically released rat calvaria cell populations. Calcif. Tissue Int. 38, 143–154 10.1007/BF02556874 3085892

[39] Michaylova V., and Ilkova P. (1971) Photometric determination of micro amounts of calcium with arsenazo III. Anal. Chim. Acta 53, 194–198 10.1016/S0003-2670(01)80088-X

[40] Bradford M. M. (1976) A rapid and sensitive method for the quantitation of microgram quantities of protein utilizing the principle of protein-dye binding. Anal. Biochem. 72, 248–254 10.1016/0003-2697(76)90527-3 942051

[41] Dimauro I., Pearson T., Caporossi D., and Jackson M. J. (2012) A simple protocol for the subcellular fractionation of skeletal muscle cells and tissue. BMC Res. Notes 5, 513 10.1186/1756-0500-5-513 22994964 PMC3508861

