# Supplementary material for: Calreticulin regulates a switch between osteoblast and chondrocyte lineages derived from murine embryonic stem cells
Source: J Biol Chem. 2020 Mar 27;295(20):6861–75. doi: 10.1074/jbc.RA119.011029 (PMC7242707; doi:10.1074/jbc.RA119.011029)
Supplement: Supporting Information [file supp_295_20_6861__index.html]

Calreticulin regulates a switch between osteoblast and chondrocyte lineages derived from murine embryonic stem cells — Calreticulin regulates osteogenesis and chondrogenesis — Calreticulin regulates a switch between osteoblast and chondrocyte lineages derived from murine embryonic stem cells — Calreticulin regulates osteogenesis and chondrogenesis — Supporting Information 

# Calreticulin regulates a switch between osteoblast and chondrocyte lineages derived from murine embryonic stem cells

## Supporting Information

- Supporting Information (to be published online) - Supplemental Figures S1-S5 and Supplemental Tables 1-2
