# Supplementary material for: Calreticulin regulates a switch between osteoblast and chondrocyte lineages derived from murine embryonic stem cells
Source: J Biol Chem. 2020 Mar 27;295(20):6861–75. doi: 10.1074/jbc.RA119.011029 (PMC7242707; doi:10.1074/jbc.RA119.011029)
Supplement: Supporting Information [file supp_RA119.011029_155778_1_supp_490093_q6xt21.pdf]

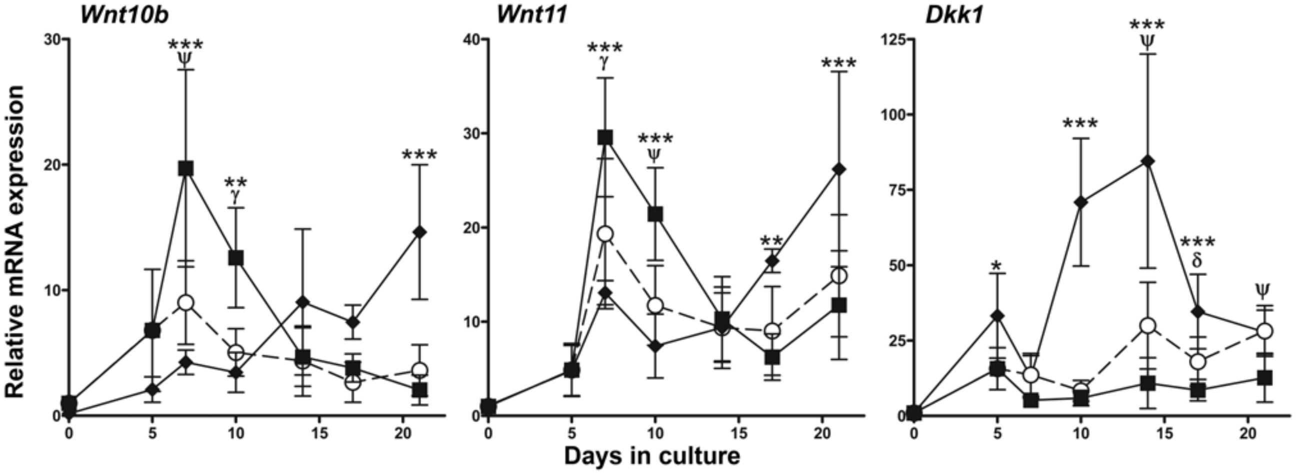

Supplemental Figure 1.
Wnt signalling pathway genes are affected by calreticulin and NFAT.
Real-time quantitative reverse transcription PCR analysis of Wnt pathway genes using RNA extracted at indicated times during the osteoblast differentiation protocol. Expression of genes was normalized to L32 house keeping gene as an internal standard and relative to transcript levels in WT cell at day 0. WT (■), and *crt*$^{-/-}$ (♦) ESCs, and WT cells treated with A-285222 (○) for days 6-17. Data expressed as means ±SD, and n of minimum 3. 2-way ANOVA analysis: Transcript levels between WT control and *crt*$^{-/-}$ ESCs (*Wnt10b*, P=0.1476, F=2.16; *Wnt11*, P=0.3861, F=0.7631; *Dkk1*, P<0.0001, F=187.8). Transcript levels in WT control ESCs treated or not with A285222 (*Wnt10b*, P=0.0014, F=11.09; *Wnt11*, P=0.0378, F=4.466; *Dkk1*, P<0.0001, F=42.5). Bonferroni post hoc analysis as indicated: *** = p<0.001; **=p<0.01; * = p<0.05; ψ = p<0.001, and γ = p<0.01; d<0.05 for DMSO-treated WT vs. A-285222-treated WT ESCs.

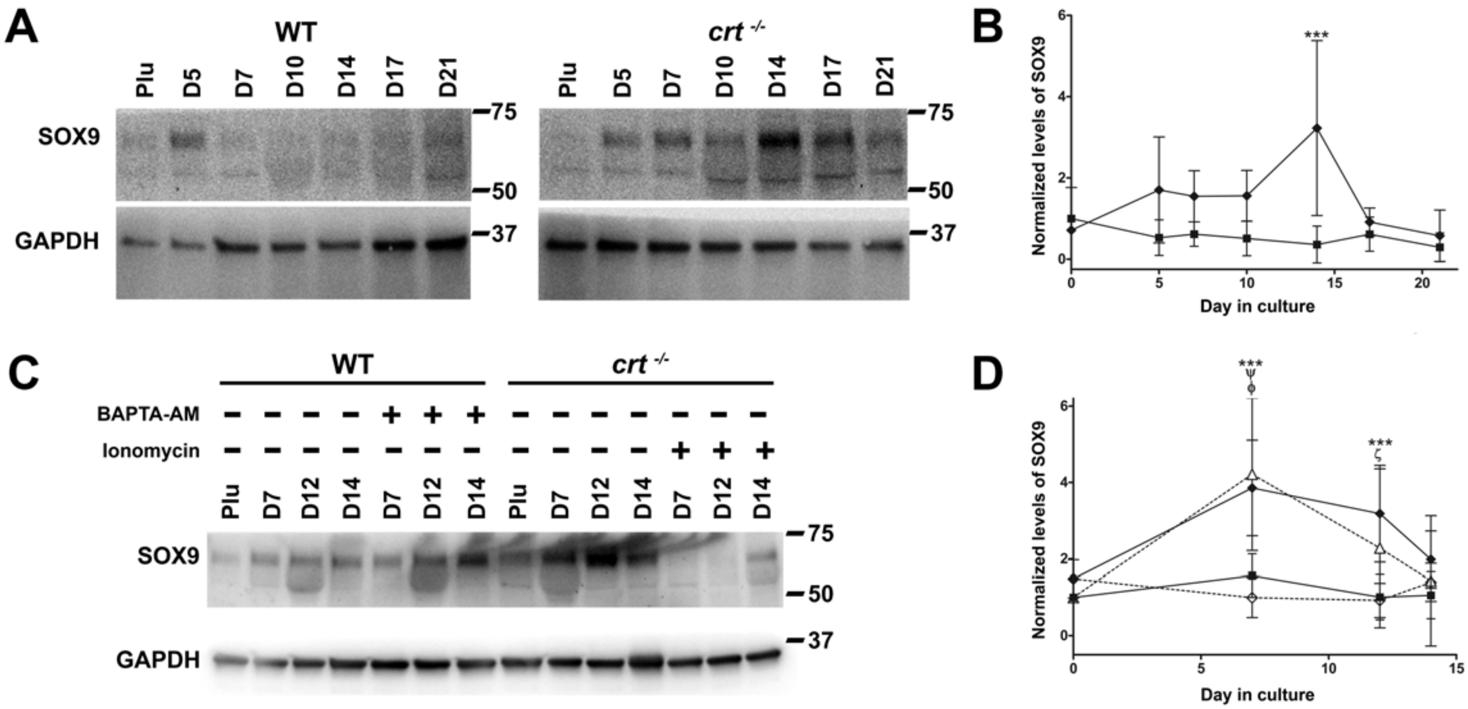

Supplementary Figure 2
SOX9 protein levels are regulated by calreticulin/Ca²⁺ during osteoblast differentiation.

(A and C) Western blot analysis of whole cell lysates harvested at indicated times from WT control (■), and *crt*-/- (♦) ESCs (B and D), or WT control, and *crt*-/- ESCs treated on days 3 to 5 with BAPTA-AM (Δ) or ionomycin (◊), respectively, during osteoblast differentiation (D). Utilized antibodies specific to SOX9, and GPADH as an internal control. (B and D) Quantitative representation of the density of the Western blot bands of SOX9 normalized to internal control GPADH. Data expressed as means ±SD, and n of minimum 3. 2-way ANOVA analysis of band densities: (B) $P<0.0001$ and $F=24.69$, and (D) $P<0.0001$ and $F=35.02$ for WT control and *crt*-/- ESCs; $P=0.0066$ and $F=8.372$ for WT control ESCs treated or not with BAPTA-AM; $P<0.0001$ and $F=29.21$ for *crt*-/- ESCs treated or not with ionomycin. Bonferroni post hoc test where indicated: ***=$P<0.001$ for WT control and *crt*-/- ESCs data sets; ϕ=$P<0.05$ for WT control ESCs treated or not with BAPTA-AM; ψ = $P<0.001$ and ζ=$P<0.01$ for *crt*-/- ESCs treated or not with ionomycin.

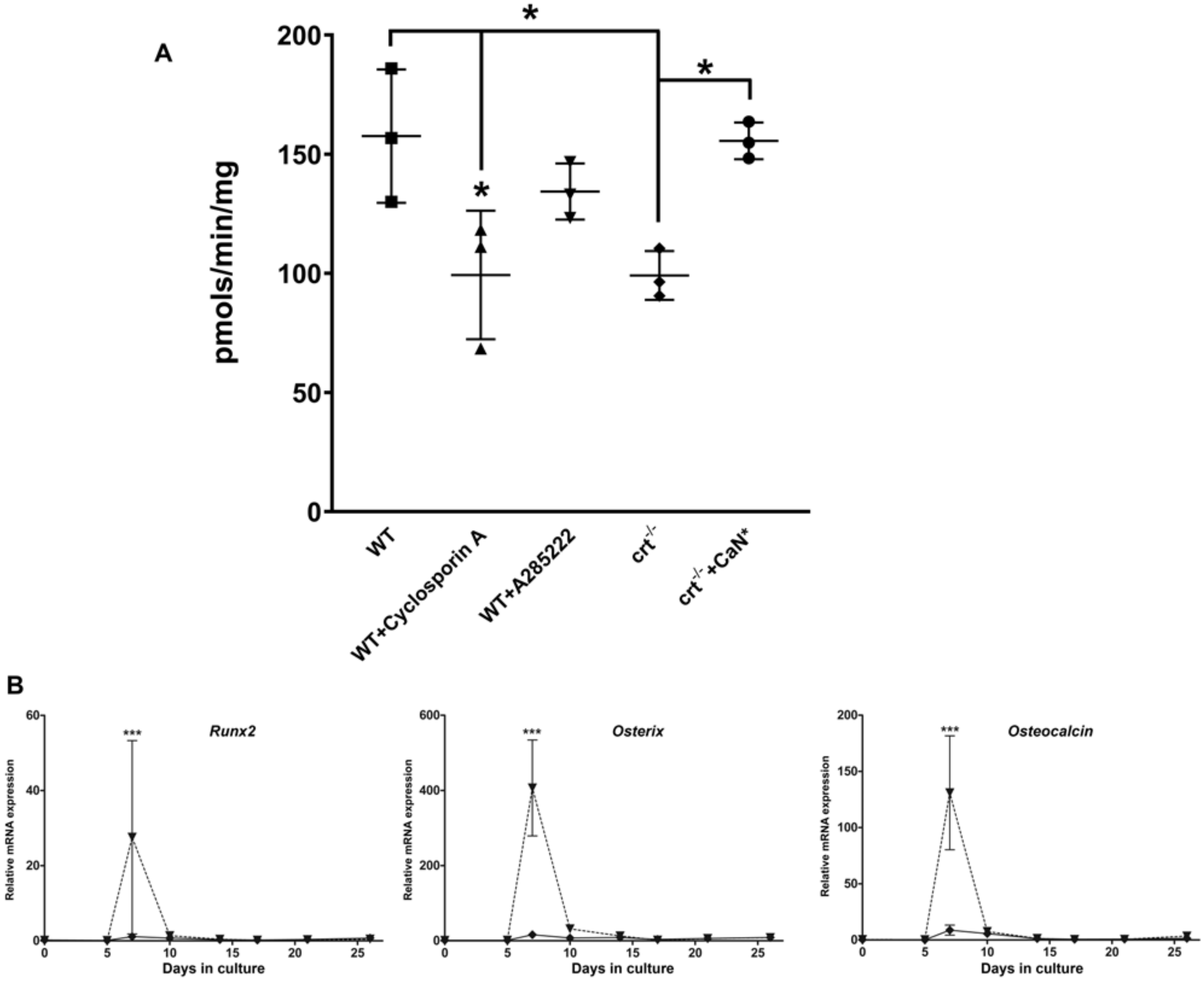

Supplemental Figure 3.
Calcineurin activity of ESCs.
(A) Radiolabelled peptide-based assay for the phosphatase activity of calcineurin from whole-cell extracts of WT cell in the presence of cyclosporin A or A-285222 as indicated or *crt*$^{-/-}$, and *crt*$^{-/-}$ +CaN* ESCs. Data expressed as means ±SD, n=3. 1-way ANOVA: P=0.0063, F=6.875. Bonferroni post hoc analysis as indicated: * = P<0.05. (B) RT-qPCR comparison of osteoblast gene levels in either *crt*$^{-/-}$ (♦), or *crt*$^{-/-}$ +CaN* (▼) ESCs at indicated time points during the osteoblast differentiation protocol. Data expressed as means ±SD, and n of minimum 3. 2-way ANOVA analysis: *Runx2*, P=0.0108, F=7.254; osterix, P<0.0001, F=70.41; osteocalcin, P<0.0001, F=43.21.
Bonferroni post hoc analysis as indicated: *** = P<0.001.

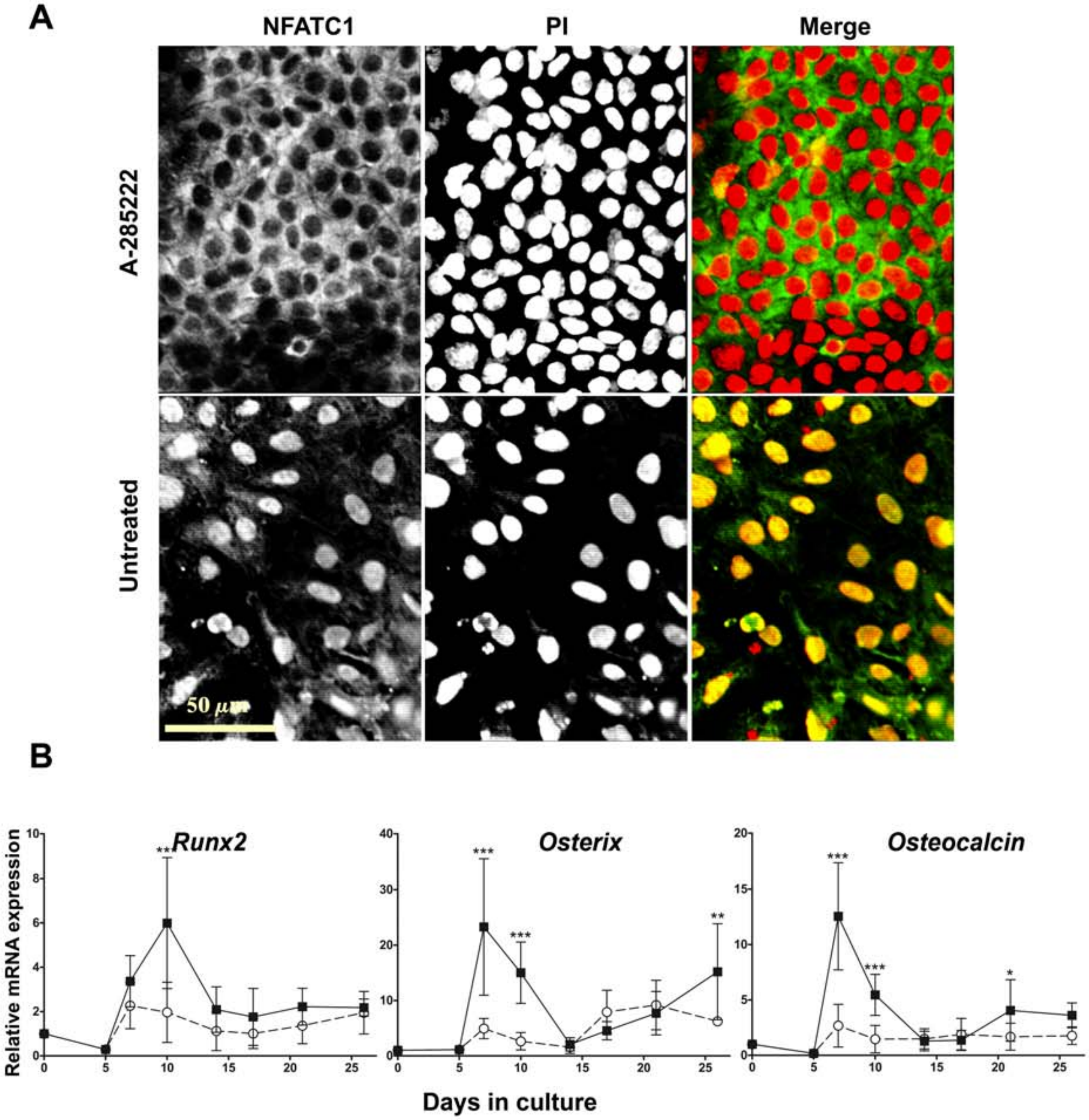

Supplemental Figure 4.
The effect of A-285222 treatment on the localization of NFATC1 in ESCs.
(A) Confocal images of an immunofluorescence localization analysis using NFATC1 antibody on WT control ESCs on day 14 of the osteoblast differentiation. Dual channel images of a single field are displayed in grey scale, of which NFATC1 localization is in the left panel and PI-labelled nuclei are in the right panel, while the RGB panel shows merged images. Scale = 50 μm. (B) RT-qPCR analysis of osteoblast genes through time of differentiation in DMSO-treated WT ESCs (■), and WT ESCs treated with A-285222 (○) for days 6 to 17. Data expressed as means ±SD, and n of minimum 3. 2-way ANOVA analysis: *Runx2*, $P<0.0001$, $F=25.67$; osterix, $P<0.0001$, $F=35.9$; osteocalcin, $P<0.0001$, $F=72.9$. Bonferroni post hoc analysis as indicated: * = $P<0.05$; ** = $P<0.01$; *** = $P<0.001$.

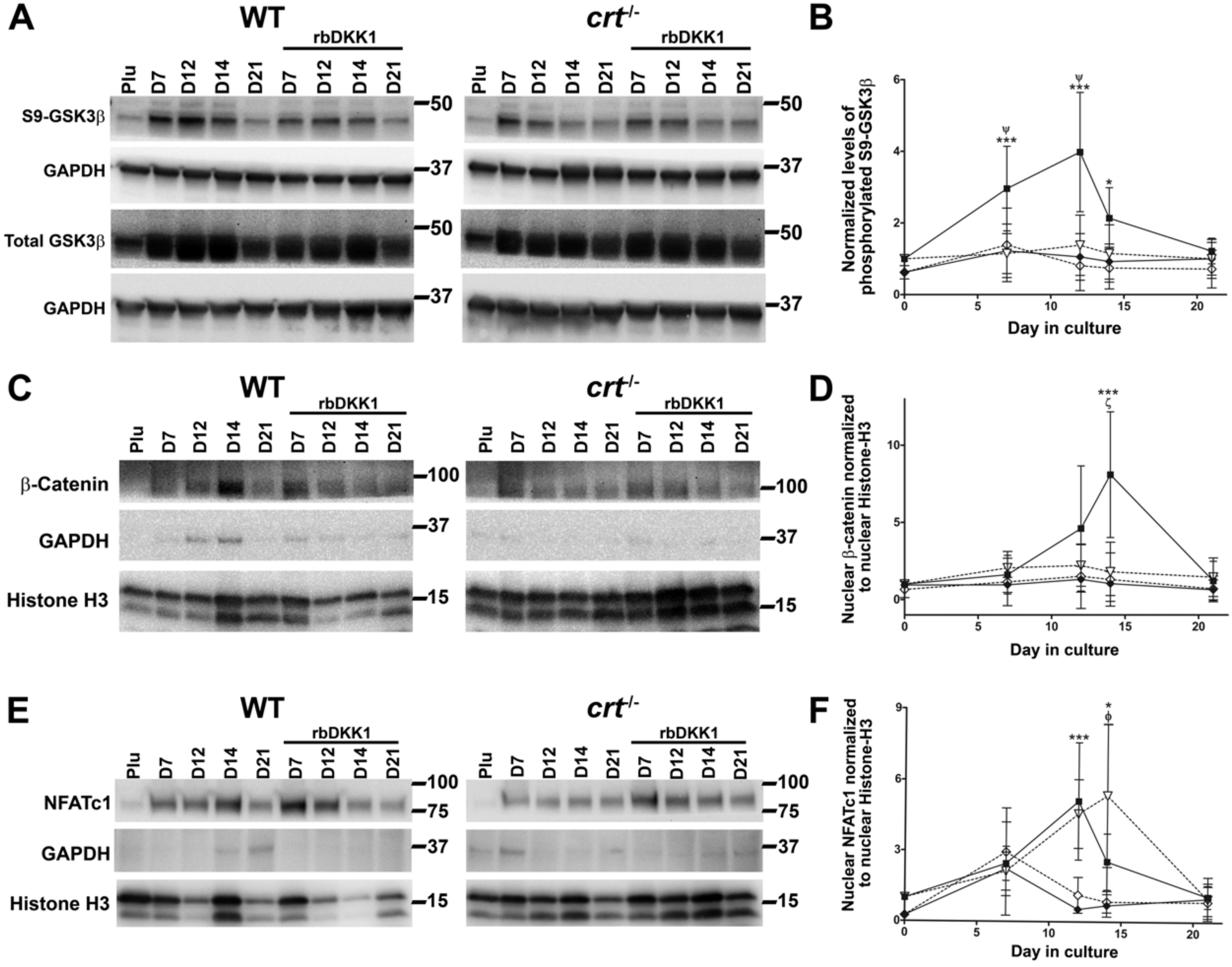

Supplemental Figure 5. Recombinant DKK1 attenuates GSK3β-deactivation, and decreases nuclear-β-catenin levels during osteoblast differentiation, but does not affect nuclear-NFATc1 levels. Western blot analysis of whole lysates or nuclear fractions extracted from WT control and *crt*$^{-/-}$ ESCs osteoblast differentiation cultures. Where indicated cultures were incubated with rbDKK1 on days 6 to 17. (A) Western blots analysis of whole lysates utilizing antibodies specific to phosphorylated serine-9 of GSK3β, or whole GSK3β, and GAPDH as internal loading standard. (C and E) Western blots analysis of nuclear fractions utilizing antibodies specific to β-catenin, or NFATc1 and histone-H3, or GAPDH as the internal nuclear, or cytosolic standard, respectively. (B, D and F) Quantitative representation of the density of the Western blot bands (WT control ESCs = ■ ; *crt*$^{-/-}$ ESCs = ♦, and WT ESCs + rbDKK1 = ∇; *crt*$^{-/-}$ ESCs + rbDKK1 = ◊). Data expressed as means ±SD, and n of minimum 3. 2-way ANOVA analysis of band densities: (B) P<0.0001 and F=54.83 for WT control and *crt*$^{-/-}$ ESC data set; P<0.0001 and F=37.55 for WT control ESCs treated or not with rbDKK1; P=0.4348 and F=0.6187 for *crt*$^{-/-}$ ESCs treated or not with rbDKK1. (D) P<0.0001 and F=22.46 for WT control and *crt*$^{-/-}$ ESC data set; P=0.0339 and F=4.875 for WT control ESCs treated or not with rbDKK1; P=0.7782 and F=0.08056 for *crt*$^{-/-}$ ESCs treated or not with rbDKK1. (F) P<0.0001 and F=24.02 for WT control and *crt*$^{-/-}$ ESC data set; P=0.3456 and F=0.9122 for WT control ESCs treated or not with rbDKK1; P=0.3622 and F=0.8511 for *crt*$^{-/-}$ ESCs treated or not with rbDKK1. Bonferroni post hoc test where indicated: *=P<0.05 and ***=P<0.001 for WT control and *crt*$^{-/-}$ ESCs data sets; Ψ=P<0.001; ζ=P<0.01 and ϕ=P<0.05 for WT control ESCs treated or not with rbDKK1.

Supplemental Table 1: nuclear localization of NFAT at D10 of Differentiation

	NFAT localization	Manders' coefficient
WT	100% nuclear	0.819
crt$^{-/-}$	7% nuclear	0.329
crt$^{-/-}$+CaN*	84% nuclear	0.756

Supplemental Table 2: Reverse transcriptase real time PCR primers.

Gene	Forward (5'-3')	Reverse (5'-3')	Source
L32	CATGGCTGCCCTTCGGCCTC	CATTCTCTTCGCTGCGTAGCC	PMID: 10617337
Runx2	CCTCTGACTTCTGCCTCTGG	TAAAGGTGGCTGGGTAGTGC	PMID: 25613029
Sp7 (Osterix)	CGCTTTGTGCCTTTGAAAT	CCGTCAACGACGTTATGC	PMID: 19448637
Ibsp	ACAATCCGTGCCACTCACT	TTTCATCGAGAAAGCACAGG	PMID: 19448637
Bglap3 (Ocn)	GCCGGAGTCTGTTCACTACC	GCGCTCTGTCTCTCTGACCT	Primer depot
Col2a1	GCAAGATGAGGGCTTCCATA	CTACGGTGTCAGGGCCAG	Primer depot
Sox9	TCCACGAAGGGTCTCTTCTC	AGGAAGCTGGCAGACCAGTA	primer depot
Agc1	CGCTCAGTGAGTTGTCATGG	GGAGCGAGTCCAACTCTTCA	Primer depot
Wnt10b	ACCACGACATGGACTTCGGAGA	CCGCTTCAGGTTTTCCGTTACC	PMID: 23900840
Wnt11	AGGCCCTCCAGCTGTTTAC	GAGGCTCTGCTCTTTGCCTT	Primer depot
Dkk1	ATGCTTTCCTCAATTTCCCC	ATGAGGCACGCTATGTGCT	Primer depot
Dscr1	TTTATCCGGACACGTTTGAA	GTGTGGCAAACGATGATGTC	Primer depot
